# Using Graph Components Derived from an Associative Concept Dictionary to Predict fMRI Neural Activation Patterns that Represent the Meaning of Nouns

**DOI:** 10.1371/journal.pone.0125725

**Published:** 2015-04-30

**Authors:** Hiroyuki Akama, Maki Miyake, Jaeyoung Jung, Brian Murphy

**Affiliations:** 1 Graduate School of Decision Science and Technology, Tokyo Institute of Technology, Tokyo, Japan; 2 Graduate School of Language and Culture, Osaka University, Osaka, Japan; 3 Machine Learning Department, Carnegie Mellon University, Pittsburgh, United States of America; Plymouth University, UNITED KINGDOM

## Abstract

In this study, we introduce an original distance definition for graphs, called the Markov-inverse-F measure (MiF). This measure enables the integration of classical graph theory indices with new knowledge pertaining to structural feature extraction from semantic networks. MiF improves the conventional Jaccard and/or Simpson indices, and reconciles both the geodesic information (random walk) and co-occurrence adjustment (degree balance and distribution). We measure the effectiveness of graph-based coefficients through the application of linguistic graph information for a neural activity recorded during conceptual processing in the human brain. Specifically, the MiF distance is computed between each of the nouns used in a previous neural experiment and each of the in-between words in a subgraph derived from the Edinburgh Word Association Thesaurus of English. From the MiF-based information matrix, a machine learning model can accurately obtain a scalar parameter that specifies the degree to which each voxel in (the MRI image of) the brain is activated by each word or each principal component of the intermediate semantic features. Furthermore, correlating the voxel information with the MiF-based principal components, a new computational neurolinguistics model with a network connectivity paradigm is created. This allows two dimensions of context space to be incorporated with both semantic and neural distributional representations.

## Introduction

Complex networks are frequently represented in the form of graphs consisting of nodes (vertices) denoting individual (or atomic) entities, and edges that link them according to information about semantic attributes or some weighting value. Graph coefficients have a long history, especially in well-developed social networks, such as the Jaccard or Simpson indexes. In cognitive linguistics and psychology, a network view can be applied to the world of language, and conceptual interrelations can be represented in graph form as a semantic network. Word association norms representing the relationship between words are a traditional and conventional object of research—such associations have undoubtedly served as valuable language resources in the construction of semantic networks.

Since the work of Galton, word association [1–4] has been used as an empirical method for observing thought processes, memory, and mental states in clinical and cognitive psychology [5–10]. Associative Concept Dictionaries (ACDs) consist of word-pair data from psychological experiments in which participants provide a semantically related response word to the given stimulus word [11–15].

At the start of the 21^st^ century, advanced techniques involving complex networks began to be applied to language corpora to enhance lexical semantic analysis. Dorow et al. [16] utilised graph clustering techniques to detect lexical ambiguity and acquire semantic classes. Tenenbaum and Steyvers [17] conducted a noteworthy study that examined the structural features of three semantic networks (the free association norms of Nelson et al., Roget’s thesaurus, and WordNet). Rising interest in complex networks is rooted in the work of Watts and Strogatz [18] and Ferrer i Cancho and Sole [19], who elucidated the “small-world” phenomenon, and especially that of Barabási and Albert [20], who suggested that the degree distributions of scale-free network structures obey a power law.

A new similarity coefficient that integrates the classical indices of graph theory with new knowledge pertaining to structural feature extraction from semantic networks represents an important advance. In addition, as there are few objective methods for treating network similarity information in the domain of corpus analysis and psychological experimentation (aside from Word Association Space [21], for example), machine learning methods in neurolinguistics may provide a new means of evaluation for semantic network computing [22–25]. Note that, despite the significance of semantic networks built on word association norms in cognitive science and psychology, no attempt has yet been made to apply any linguistic graph information to human neural activity data recorded during conceptual processing.

This idea has great potential in light of a study reported by Mitchell et al. [26] using a large corpus of web text (the Google Web 1T 5-gram Collection). They proposed a computational model that allows the functional magnetic resonance imaging (fMRI) activity associated with thinking about arbitrary nouns to be predicted. The underlying theory is that the neural basis of the semantic representation of specific nouns is related to the distributional properties of those words in a broad-based corpus. Recently, the model of Mitchell et al. has been extended to use crowd-sourced judgments of semantic properties [27] or broader corpora [28–31].

From this perspective, a *computational neurolinguistics* model that utilises graph theory might be feasible if we could create a set of intermediate semantic features (and their weights) by applying appropriate graph coefficients for complex networks built from a small dataset of word association norms. Computational neurolinguistics is an emerging research area that aims to integrate computational linguistics and cognitive neuroscience to better understand word semantics. It takes advantage of machine learning methods to mediate datasets from neural recordings and language corpora (cf. https://sites.google.com/site/compneurowsnaacl10/). The advantage of using a graph-form representation for ACDs is that we can compute the distance or similarity coefficient between any two words from a minor lexical dataset based on the degree (the number of links held by one vertex), the degree distribution (the probability distribution of the degrees over a graph), or the shortest path information specific to a complex network (minimum number of steps from one vertex to another).

In this article, we propose a new similarity index that is indicative of various characteristics of semantic networks, regardless of size and complexity. Furthermore, the semantic space formed by applying this similarity index to word association norms might be different from those based on co-occurrence pattern information derived from the usual lexical corpora. The n-grams extracted from a web document collection could effectively simulate the fMRI neural data from a property generation task performed on word stimuli. It is also important to determine whether semantic network information given by applying graph theory to ACDs would be effective in predicting the activity of the human brain.

Tapping into the other paradigm of complex networks, we propose an original use for neuroimaging studies using fMRI. Previously, functional connectivity MRI (fcMRI) [32–43] has been employed as an intriguing technique for uncovering chains of voxels (pixels with volume as units of neuro-imaging data) that simultaneously fire under particular task-driven or resting conditions. As a variant of fcMRI, we describe a system of informative voxels as vertices within a neural circuit that is correlated with semantic network information derived from a dataset of word association norms.

## Materials and Methods

### Definition of MiF

In this section, we introduce the Markov-inverse-F measure (MiF), a new definition of distance on a graph. MiF improves the conventional Jaccard and Simpson indices, and reconciles both the geodesic information (random walk) and co-occurrence adjustment (degree balance and correlation).

**Fig 1:**
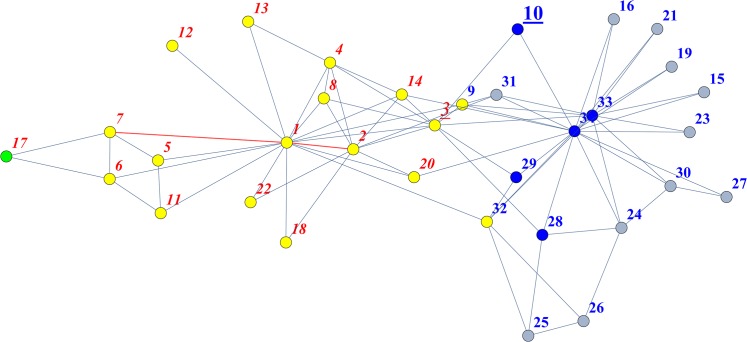
Friendship network of Zachary’s famous “Karate Club”. There is one shortest path between vertices 2 and 7 (red edges), with a step length of 2. It follows from the sum of the elements in the second and seventh rows (or columns) of the second power of the adjacency matrix that there are 52 and 25 two-step paths starting from vertices 2 and 7, respectively. Thus, the Jaccard similarity between them is calculated as (52+25)^−1^ = 0.012987, if we take into account all of the steps starting from each of the two vertices that have a step length of 2. In this figure, the yellow nodes are reachable in two steps from both vertices 2 and 7, whereas, under the same path condition, the blue nodes can only be reached from vertex 2, and the green node can only be reached from vertex 7. In addition, these two vertices have a Simpson coefficient of 25^−1^ = 0.04 and a MiF value of 0.0185583. It is widely known that the friendship network among the Karate club members was split into two factions. According to the degree to which the final attachments to each faction match with the results of graph clustering, it is possible to evaluate the effectiveness of the clustering technique (based on an adjacency matrix) for simulating the social relationships. The two factions are represented here by the vertex labels with red italic font (one group composed of vertices {1, 2, 3, 4, 5, 6, 7, 8, 11, 12, 13, 14, 17, 18, 20, 22}) and those with blue bold font (the other group of {9, 10, 15, 16, 19, 21, 23, 24, 25, 26, 27, 28, 29, 30, 31, 32, 33, 34}). Misclassification always occurred by binding vertices 3 and 10 at early stages when the Jaccard index, Simpson index, and MiF with the default *β* value (0.5) were applied to the hierarchical graph clustering of this network. With a small value of *β* (for example, 0.01), which can reflect the asymmetrical roles played by the two agents in terms of connectivity, MiF predicts the composition of the two factions with 100% accuracy. For further details, see S2 Program. This figure was created using Mathematica 8.

To give the co-occurrence adjustment, it is known that the Jaccard similarity can be intuitively formulated as

$$\frac{\left|A\cap B\right|}{\left|A\cup B\right|}$$ (1)

for two sets *A* and *B*. Indeed, for two vertices, this index is usually computed as

$$\frac{\left|N(\text{a})\overset{}{\underset{}{\cap }}N(b)\right|}{\left|N(a)\overset{}{\underset{}{\cup }}N(b)\right|},$$ (1)'

where *N*(*a*) denotes the set of all neighbours of vertex *a*. To enhance the accuracy with which the distance between remote nodes is evaluated, we extend the interpretation of expression (1) such that the numerator is the distance of the shortest path connecting vertices *a* and *b*. The denominator in (1) is the sum of the degrees of vertices *a* and *b*, or, in some cases, all of the steps starting from these vertices that have an identical step length. In this article, we adopt the latter definition for the denominator, and set the step length equal to the shortest path between *a* and *b* in the numerator. Fig 1 illustrates this coefficient using the friendship network of Zachary’s famous “Karate Club” [44].

Certain disadvantages of the Jaccard similarity have been described. For example, it can produce values that are too small and not intuitively plausible. This is because the denominator for normalisation, i.e. the cardinality of the union of two sets, is often too large [45–46]. To compensate for this perceived weakness, the Simpson index was introduced. Given by

$$\frac{\left|A\cap B\right|}{Min(|A|,|B|)},$$ (2)

this index tends to return a larger similarity score for connections with a small-degree vertex, which would bias the degree imbalance of the two vertices of interest. In (2), |*A*| and |*B*| represent the number of paths starting from vertices *a* and *b*, respectively. Note that the step length for |*A*| and |*B*| in the denominator of (2) is the same as that for the numerator, namely, the shortest path between the two nodes.

Inspired by the Simpson index, we generalise this to reflect multiple features of a network. Our idea consists of modifying the denominator using the weighted harmonic mean

$$H_{\beta }(a,b)=\frac{1}{\frac{1-\beta }{a}+\frac{\beta }{b}}=\frac{ab}{\beta a+(1-\beta )b}$$ (3)

of all *i*-step paths leaving the two vertices. Our new distance for two sets *A* and *B* is then

$$\frac{\left|A\cap B\right|}{H_{\beta }(|A|,|B|)}.$$ (4)

Thus, the weight of the free parameter 0 < *β* < 1 enables the flexible adjustment of the magnitude of the denominator in a similar manner to the F-measure (in the field of information retrieval for making trade-offs between recall and precision). By this means, our graph index can reflect the value of the degree correlation [47–49], which might be important for some network settings, especially in weighting the vertices. For example, when there is no degree correlation, as in the Barabási-Albert model, we could assign a significantly high (or low) *β*-value to vertices with particularly high (or low) degrees. Adjusting *β* has a significant effect on the results of simulations such as graph clustering. With a small *β* (for example, 0.01), MiF enables us to attain perfect accuracy when applied with Ward’s minimum variance dissimilarity to simulate the fragmentation of the famous Karate Club. In contrast, the Jaccard index, Simpson index, and MiF with the default *β* value (0.5) all fail to correctly assess the affiliation of a specific vertex. Further details are provided in Fig 1.

In addition to evaluating the co-occurrence information, our method takes into account the geodesic-based idea of a random walk [50–51]. Between vertices x and y, it is natural to consider that a greater number of connecting paths indicates a closer relationship in the graph. However, the number of shortest path lengths greater than one step, or that of bypasses including redundant loops, can be an important factor [21, 52–54] if the other weight parameter is configured for the path steps of a random walker on a graph. The values of this parameter for *i*-steps, *α*_*i*_, should decrease with similarity in accordance with the procession of a random walk.

This combination of a random walk transition (Markov process) on a graph and a harmonic mean for information retrieval explains the name of our new similarity coefficient, the Markov-inverse-F Measure, or MiF.

We now present some notation and definitions that we use to describe a complex network:

*A*: an m*m adjacency matrix for a graph having *m* vertices,

*A*_*x*,*y*_: the (*x*, *y*)-th element of *A*,

$A_{x,y}^{p}$: the (*x*, *y*)-th element of the path matrix *A* ^*p*^,

$S_{x,y}^{\left(i\right)}=A_{x,y}^{i}$: the number of paths (routes) connecting vertices *x* and *y* with *i* steps,

$P_{x}^{\left(i\right)}=\sum_{c=1}^{m}A_{x,c}^{i}$: the number of paths (routes) starting from vertex *x* that have a length of *i* steps.

Regarding *α*_*i*_, we implement the constraints

$$0<\alpha_{i}<1,\alpha_{1}>\alpha_{2}>\ldots ,\sum_{i=1}^{\gamma }\alpha_{i}=1$$ (5)

for the scaling between 0 and 1 to be imposed to MiF, as the values of this parameter are reduced with an increase in path steps. Let *γ* denote a small integer delimiting the maximum number of steps *i*. This can be determined by the extent of *small-worldness* [18], and is usually less than around ten for a graph built on a language corpus (cf. [17]: the maximum average shortest path length recorded in WordNet is 10.56). Values of *α*_*i*_ can be provided empirically by the following reference coefficient list, whose number of elements is set to this provisory limit.

Given

c={1.,1.61803,1.83929,1.92756,1.96595,1.98358,1.99196,1.99603,1.99803,1.99902}

as the maximum real roots of $\sum_{i=1}^{k}{\left(\frac{1}{x}\right)}^{i}=1$ for all integers *k* (0 < *k* ≤ 10), the expressions *α*_*i*_ = *c*(*γ*)^−*i*^ and (5) always hold. For instance, with *γ* = 2 as the maximum step length, we have:

$$\sum_{i=1}^{\gamma }\alpha_{i}=\sum_{i=1}^{2}c{\left(2\right)}^{-i}=1.61803^{-1}+1.61803^{-2}=1;$$

with *γ* = 4:

$$\sum_{i=1}^{\gamma }\alpha_{i}=\sum_{i=1}^{4}c{\left(4\right)}^{-i}=1.92756^{-1}+1.92756^{-2}+1.92756^{-3}+1.92756^{-4}=1,$$

and with *γ* = 10,

$$\sum_{i=1}^{\gamma }\alpha_{i}=\sum_{i=1}^{10}c{\left(10\right)}^{-i}=1.99902^{-1}+1.99902^{-2}+1.99902^{-3}+1.99902^{-4}+1.99902^{-5}+1.99902^{-6}+1.99902^{-7}+1.99902^{-8}+1.99902^{-9}+1.99902^{-10}=1$$

In addition, a constant value *β*, where 0 < *β* < 1, is given to define the weighted harmonic mean in (4). For the purpose of illustration, we set *β* = 0.5 as the default for treating all vertices with equal weight. Based on these parameters, MiF is formulated as

$$\begin{array}{l} D_{xy}=\sum_{i=1}^{\gamma }\frac{\alpha_{i}S_{x,y}^{\left(i\right)}}{H_{\beta }(P_{x}^{\left(i\right)},P_{y}^{\left(i\right)})}=\sum_{i=1}^{\gamma }\frac{\alpha_{i}S_{x,y}^{\left(i\right)}(\beta P_{x}^{\left(i\right)}+(1-\beta )P_{y}^{\left(i\right)})}{P_{x}^{\left(i\right)}P_{y}^{\left(i\right)}}\\ =\sum_{i=1}^{\gamma }\frac{\alpha_{i}A_{x,y}^{i}(\beta \sum_{c=1}^{m}A_{x,c}^{i}+(1-\beta )\sum_{c=1}^{m}A_{y,c}^{i})}{\sum_{c=1}^{m}A_{x,c}^{i}\sum_{c=1}^{m}A_{y,c}^{i}} \end{array}$$ (6)

It can be ascertained that 0 ≤ *D*_*xy*_ < 1 is true in any case. S1 Program implements functions for computing the MiF, Jaccard, and Simpson coefficients between any vertices in a network whose adjacency matrix is provided as a sparse array.

### Application of MiF

As noted in the Introduction, ACDs contain word-pair data obtained from psychological experiments in which the participants are typically asked to provide a semantically related response word that comes to mind when presented with a stimulus word. The Edinburgh Word Association Thesaurus of English (EAT, [1]) is a typical English-language ACD, and is well-balanced though small in size (approximately 3 MB). The characteristic aspect of this database is that word association norms were collected by growing the network from a nucleus of words to obtain further responses. Such a chain association gave rise to linkages among seemingly unrelated words with diverse semantic relationships.

**Fig 2:**
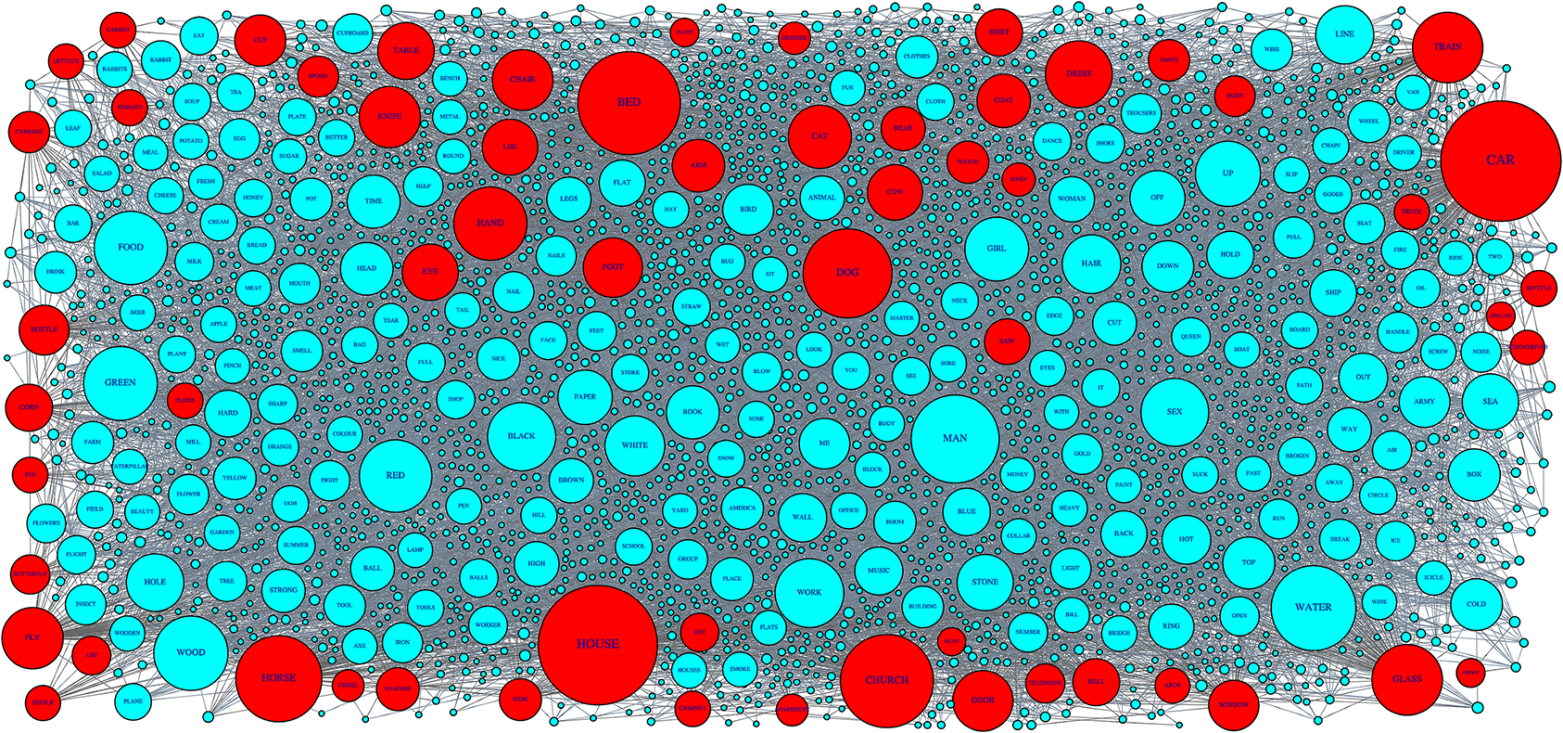
EAT subgraph around the lexical stimuli used in by Mitchell et al. This graph is composed of the 60 fMRI nouns used by Mitchell et al. [26] (red circles) and the 2708 in-between words (blue circles) linking them on the shortest path routes in the EAT semantic network. The magnitude of the radius for each vertex corresponds to the degree value. This visualisation was made with R using the igraph package.

In this research, we extract a subgraph from the EAT that connects all 60 nouns used as stimulus items in the fMRI experiments of Mitchell et al. [26] (Fig 2). These fMRI nouns are classified into 12 semantic categories (animals, body parts, buildings, building parts, clothing, furniture, insects, kitchen items, tools, vegetables, vehicles, and other man-made items), each including five nouns. EAT contains all of these fMRI nouns, except ‘CELERY’ and ‘REFRIGERATOR’, so ‘CABBAGE’ and ‘FRIDGE’ are instead selected as synonyms for these absent nouns. This non-directed and non-weighted subgraph (see S1 and S2 Datasets) has 2768 vertices (60 fMRI nouns plus 2708 in-between words), a connection rate of 0.005, mean degree of 7.23, and clustering coefficient of 0.042. The maximum and mean shortest path lengths between the fMRI nouns and the in-between words are 6 (so we set *γ* = 6) and 4.09, respectively. The degree distribution follows a clear power law (or, more specifically, Zipf’s law) [20,17].

For our computational neurolinguistics modelling, we applied our MiF Mathematica program (see S1 Program) with β = 0.5 and γ = 6 as the maximum shortest path length to the subgraph described above. We then measured the distance between each of the 60 fMRI target nouns and the 836 in-between words with degree greater than 5. This threshold was conveniently set for exemplary purposes, taking into account the importance of the words. Further, using the ‘princomp2.R’ routine (http://aoki2.si.gunma-u.ac.jp/R/src/princomp2.R), we ran a principal component analysis (PCA) on the 60 × 836 MiF-based distance matrix (S3 Dataset). This R function enables the PCA of a data matrix in which the column dimension is greater than the row dimension. Sixty principal components were extracted, and a 60 × 60 PC-score matrix representing the essential information about a partial semantic network of EAT was formed.

All the principal components extracted from the MiF-based distance matrix are identified by the short-hand notation ‘MiF-PC’, with a number in descending order of eigenvalues. Because each MiF-PC is a complex, multifaceted semantic entity, it would be difficult (besides a few exceptions, such as PC3 signifying “sex”) to unify all possible interpretations under a single heading. Thus, we instead give certain statistical information. For instance, the combination of the most contributory fMRI noun with the largest principal component score is enclosed in single quotation marks, and the most constitutive semantic features with the largest principal component loadings are written in italic font (e.g. MiF-PC1: ‘train’-*RAIL-TRAVEL-OMNIBUS-ENGINE-BUS…*). Detailed information about the MiF-PCs derived from EAT can be found in the S1 Table.

### Evaluation of MiF

**Fig 3:**
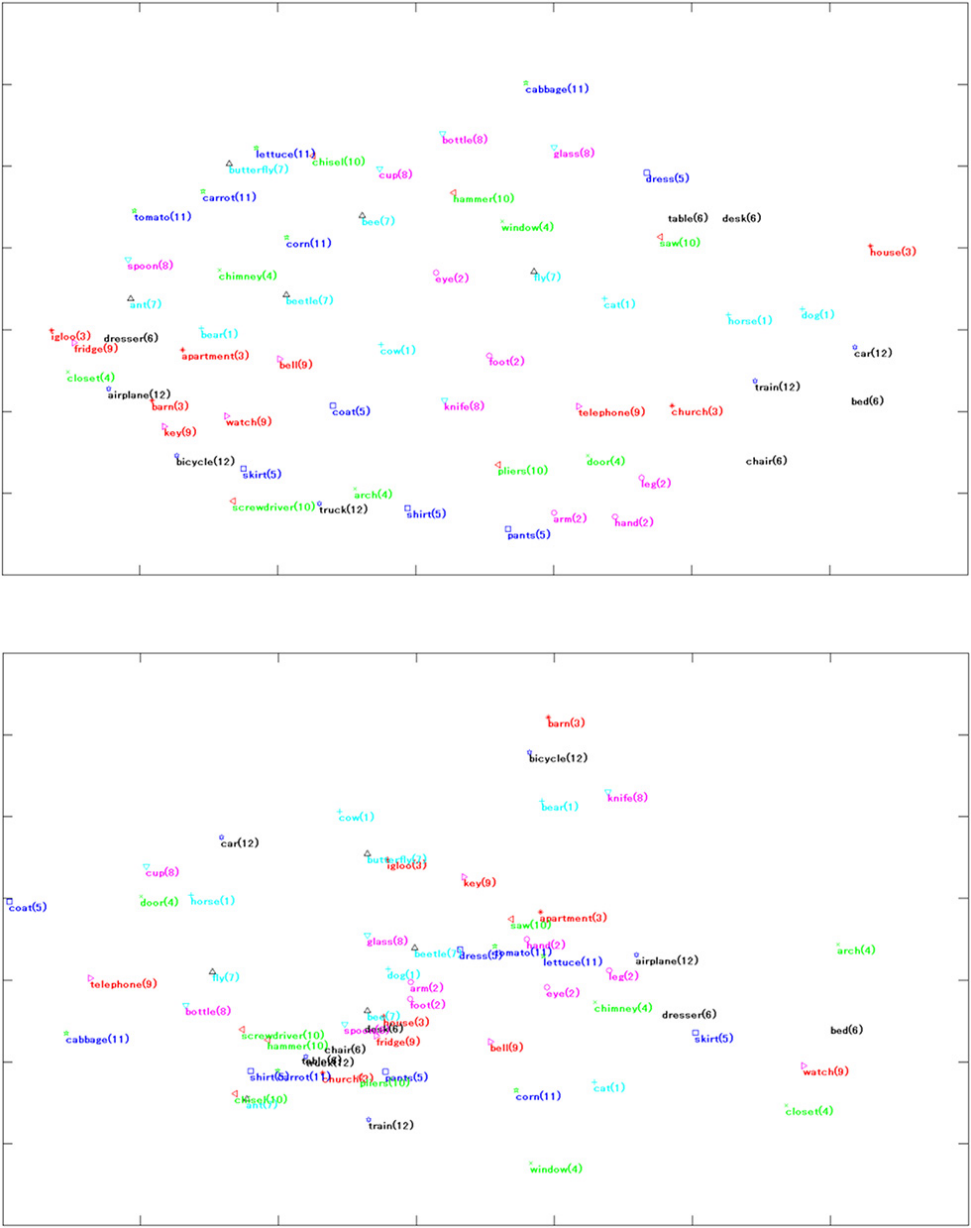
MDS results for the two distance matrices on the 60 fMRI nouns. We compared the PC-score matrix of MiF-EAT (top) with the co-occurrence matrix of Mitchell et al.’s model from the Google Web 1T 5-gram collection (bottom). The natural numbers attached to the nouns represent the semantic categories (animals: cyan-(1), body parts: magenta-(2), buildings: red-(3), building parts: green-(4), clothing: blue-(5), furniture: black-(6), insects: cyan-(7), kitchen items: magenta-(8), man-made objects: red-(9), tools: green-(10), vegetables: blue-(11), and vehicles: black-(12)). The computation and visualisation were made using Statistics Toolbox and Matlab.

It is worth noting that the semantic space underlying the 60 fMRI nouns of Mitchell et al. reflects some conceptual relationships suited to word association when using MiF applied to EAT. Fig 3 shows the results of multi-dimensional scaling (MDS) applied to the 60 × 60 MiF-EAT PC-score matrix and the 60 × 25 co-occurrence probability matrix of Mitchell et al.’s original model computed from the Google Web 1T 5-gram Collection. In MDS, each of the fMRI nouns is assigned coordinates in each dimension of the between-object distance matrix, showing the level of similarity. Some words belonging to different semantic categories become close to each other, and this closeness can be interpreted as a type of derived *metonymic* relationship. Contiguity (‘apartment’ and ‘bell’), target objects (‘key’ for ‘barn’ and ‘apartment’), intended or unintended uses (‘pants’ and ‘arm’, ‘hand’, ‘leg’; ‘window’ and ‘hammer’), mediated associations (‘igloo’ and ‘fridge’ through ‘cold’ or ‘icy’), and so on can be retrospectively construed as reasons for affinity (even a lexical association at the level of collocation (‘cup’–‘chisel’) might be produced *ex-post facto*). As for the MDS map representing the co-occurrence matrix between the nouns and the 25 basic verbs for the original Mitchell et al. model, some categories (body parts, tools) have a tendency to conglomerate at the centre, and metonymic ex-post interpretation was not as easy on the periphery as the MDS map for MiF-EAT lexical information.

In light of the original modelling of Mitchell et al., this MiF-based association matrix played the role of *f*_*i*_(*w*) in the following expression, proposed by [26]:

$$y_{v}=\sum_{i=1}^{n}c_{vi}f_{i}(w),$$ (7)

that is, a matrix recording the value of the *i* ^*t*h^ intermediate semantic feature (in our case, principal component) for word *w*. We adopted the distance information matrix instead of using the normalised co-occurrence frequency of the stimulus noun with each of 25 basic verbs, because Mitchell et al. used a text corpus consisting of over a trillion tokens (http://www.cs.cmu.edu/~tom/science2008/semanticFeatureVectors.html).

For the other terms in (7), *y*_*v*_, the predicted activation at voxel *v* for word *w*, was taken from the fMRI datasets obtained by Mitchell et al. from nine participants (http://www.cs.cmu.edu/afs/cs/project/theo-73/www/science2008/data.html). In this experiment, nine participants (P1–P9) were requested to execute a property generation task for each of 60 nouns (with a 3 s stimulus period), and then rest for a period of 7 s with a fixation mark. fMRI scans were performed using an echo planar imaging sequence with a 1000 ms repetition time, and for six different stimuli presentation orders.

The scalar parameter *c*_*vi*_ was computed by the algorithm of Mitchell et al. For the details on the experimental settings, we fundamentally adhered to Mitchell et al., using the stability score over the runs for each voxel to select 500 features (top voxels) and the leave-two-out cross-validation procedure for the machine learning. For each participant’s fMRI dataset, the leave-two-out procedure was iterated 60! / (59! × 2!) = 1770 times, leaving out each of the possible word pairs for testing. Each item pair for evaluation was used to compute the cosine similarity between the predicted and actual fMRI scans. The expected accuracy in matching the two left-out words to their left-out fMRI images is 0.50 if the matching is performed at chance levels. According to the permutation test of Mitchell et al., observing an accuracy of 0.62 or higher for the within-subject decoding would be statistically significant at P < 10^−11^.

## Results and Discussion

### Methodological comparison

**Fig 4:**
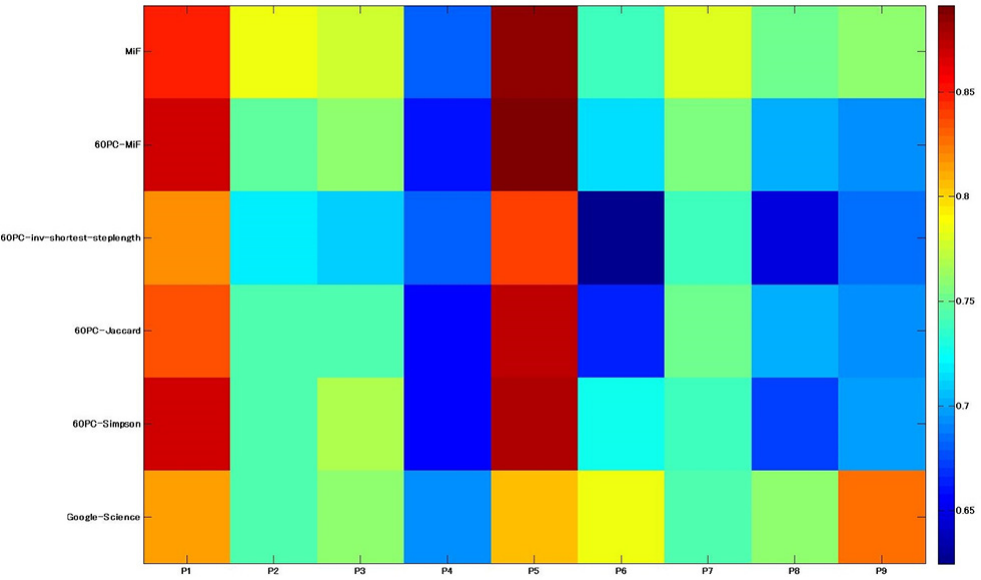
Heatmap representing the subject-wise decoding accuracy. This result was obtained under the two MiF-EAT conditions (836 words and 60 principal components), inverse shortest path step lengths, Jaccard/Simpson indices for subsequent PCA, and the replicated results of the Google-Science paper research of Mitchell et al.[26].

**Fig 5:**
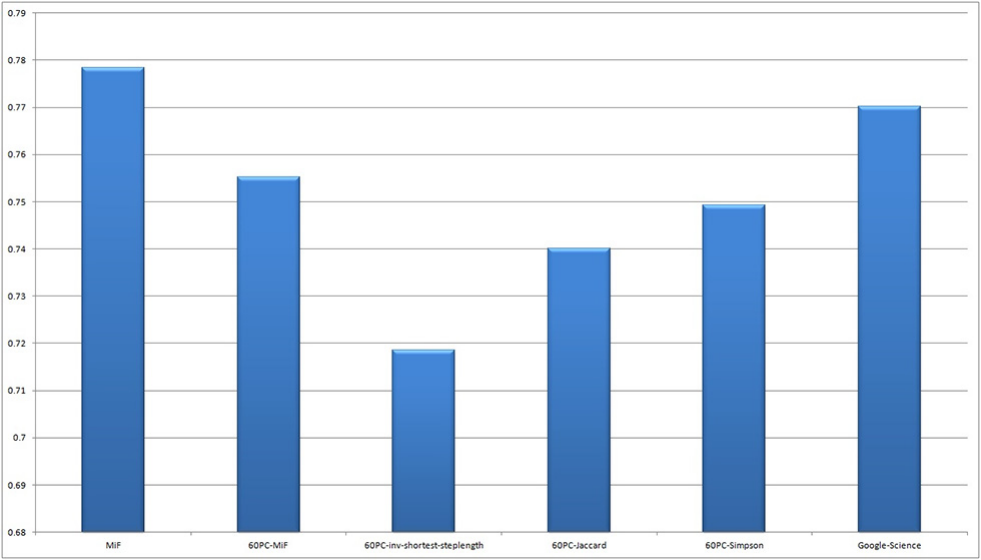
Mean discrimination accuracies obtained from the participants of Mitchell et al.'s research [26]. These results were obtained under the two MiF-EAT conditions (836 words and 60 principal components), inverse shortest path step lengths, Jaccard/Simpson indices for subsequent PCA, and the replicated results of the Google-Science paper research [26].

**Fig 6:**
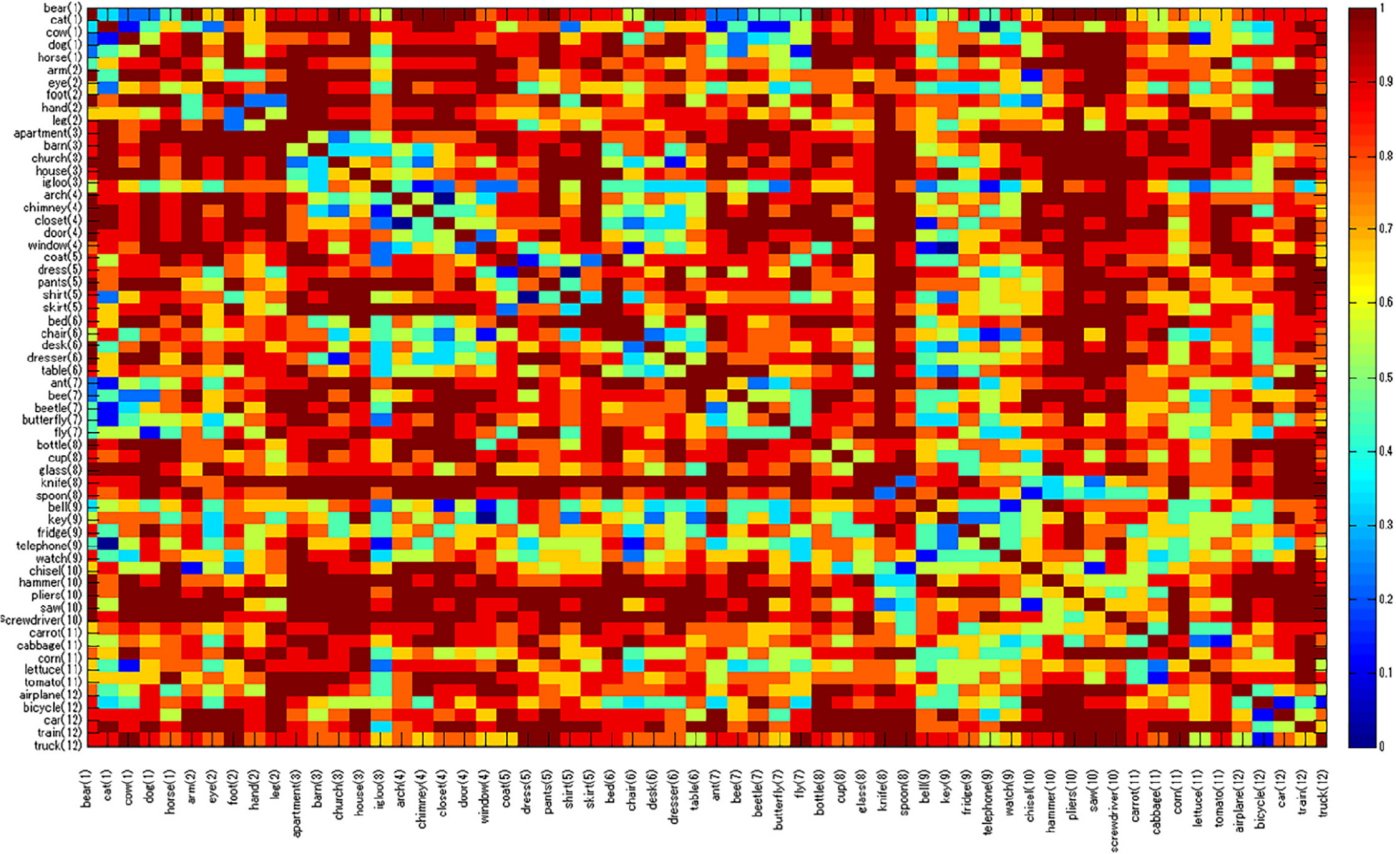
Item-wise confusion matrix from the participants of Mitchell et al.’s research [26]. The result was obtained from 60 principal components of MiF-EAT and averaged over all nine participants. The point at (row *i*, column *j*) shows the proportion of participants whose datasets allowed us to derive a correct match between the predicted noun *i* and the observed noun *j*. The number following each item name corresponds to one of these conceptual categories: (1) animals, (2) body parts, (3) buildings, (4) building parts, (5) clothing, (6) furniture, (7) insects, (8) kitchen items, (9) man-made objects, (10) tools, (11) vegetables, and (12) vehicles.

We applied MiF to the EAT subgraph, and adopted the stability score to construct graph-based models from Mitchell et al.’s fMRI datasets. The precision of our decoding models was P1: 0.85, P2: 0.79, P3: 0.78, P4: 0.68, P5: 0.89, P6: 0.74, P7: 0.78, P8: 0.75, and P9: 0.76 (mean: 0.78). The corresponding results with the 60 principal components were P1: 0.87, P2: 0.75, P3: 0.76, P4: 0.66, P5: 0.89, P6: 0.72, P7: 0.76, P8: 0.70, and P9: 0.69 (mean: 0.76). The original Mitchell et al. study recorded accuracies of P1: 0.81, P2: 0.74, P3: 0.76, P4: 0.69, P5: 0.81, P6: 0.79, P7: 0.74, P8: 0.76, and P9: 0.82 (mean: 0.77). We also computed predictive models with 60 principal components extracted from the distance matrix using the inverse shortest path step lengths (mean: 0.72), Jaccard index (mean: 0.74), and Simpson index (mean: 0.75) considering the geodesic information between nodes. A non-parametric Wilcoxon signed rank test was performed between the MiF modelling result and the closest one based on the Simpson index, both with 60 principal components. The difference was found to be highly significant (p = 7.6600e-04), and MiF outperformed the other graph similarity coefficients. Figs 4 and 5 compare the participant-wise decoding accuracy and the mean discrimination accuracy of the two MiF-based EAT analysis models, inverse shortest path step lengths, Jaccard/Simpson indices for subsequent PCA, and the replicated Mitchell et al. results with the Google 5-grams Collection. Fig 6 represents an item-wise confusion matrix generated as a result of cross-validating our decoding model trained with the 60 MiF-based principal components and averaged over all nine participants. The precision in discriminating nouns is generally good, despite a slight penalty in the within-category comparisons and the cross-category ones involving the nouns of man-made objects.

The advantage of MiF as a graph-based similarity coefficient lies in certain characteristic traits, which we now discuss. This graph-theoretical method integrates both *geodesic knowledge* (given by a random walk) and a *strength relationship* (expressed by the degree balance) from a complex network into a convenient mathematical formula. It assimilates fine-grained information about the mutual relationship between nodes, and is effectively a medium for a two-fold distributional representation of conceptual processing. The significance of MiF is underscored in terms of its predictive modelling ability across multiple research domains. A semantic network extracted from a database of word association norms (ACD) might reflect, and indeed track, the intellectual process through which corpus data grow in a chain association from a nucleus set of words. Through the intermediate semantic features shared by words in the ACD semantic network, MiF provides a good weight matrix for predicting the fMRI brain activity that might partially represent this intellectual process in another psychological experiment.

### MiF-based neuro-computational networks

Thus far, we have considered a graph-theoretical analysis through a similarity metric applied to word association norms as a source of lexical co-occurrence networks. As such, this metric might indeed be circumscribed to the semantic distance between words at the ACD level. However, an approach whereby connectivity information could be mathematically formalised may also be effective for deriving components (as neural correlates) from the patterns of fMRI signal changes detected during the processing of word senses.

This valuable insight prompts us to envision another graph-form for information in brain regions that are supposed to serve the process of conceptual association. Further details concerning this methodology, partly inspired by the ideas of fcMRI [32–43], are fully demonstrated in the S1 Text. To integrate these linguistic and physiological networks, we correlate *selected features* in the machine learning of fMRI signals (known as multi-voxel pattern analysis or MVPA, see [26, 31, 55–65]) to *semantic features* for fMRI stimulus nouns, which are treated as objects of natural language processing.

This modelling involves detecting, with respect to these nouns, a subset of informative voxels (as “*neuro-anatomical features*”) that elicit a neural activation pattern that is significantly homologous to each MiF principal component vector (derived as “*lexico-semantic features*”). We set a threshold for the pairwise Pearson correlations between these two features at 0.330104 (p < 0.01) in accord with the no-correlation test for a dimension size of 60 (equal to the number of fMRI stimulus nouns), and created a participant-wise bipartite graph between MiF principal components and important voxels (see S1 Text, Section I and S1 Fig). Our discussion here is confined to the theoretical implications of superimposing these separate feature layers in the context of *computational neurolinguistics*.

We address the issue of whether and how such a twofold modelling can incorporate aspects of *distributional representations* in a paradigm of network connectivity. The distributional representation implies the following propositions in different rubrics: the meaning of a word is defined as a set of properties or features specified in various views and contexts (see literature reviews in [66–70]); activation, even by thinking of a single word, is scattered across the whole brain [22, 25, 30–31].

**Fig 7:**
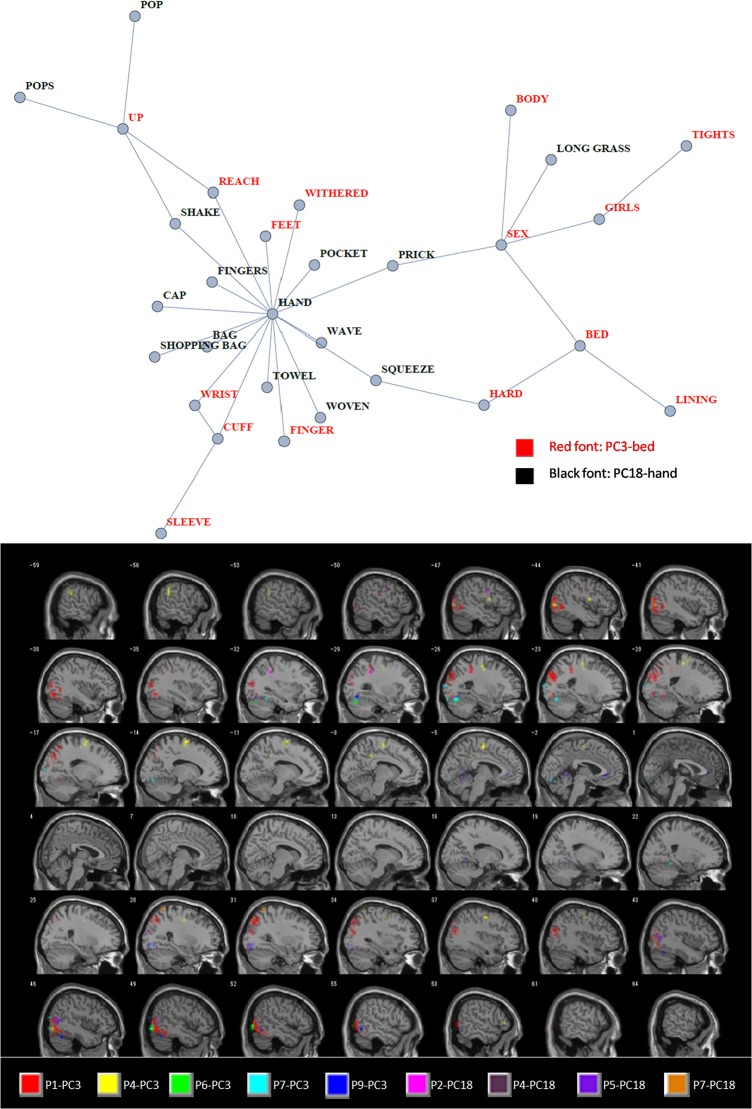
Example of conceptual association overlaid on brain images representing its neural context. Top: lexical adjacency graph extracted from the semantic network of EAT (Fig 2). This represents MiF Principal Components (MiF-PCs) 3 (red labels) and 18 (black labels) with the fMRI nouns having the largest principal component scores (“bed” and “hand”, respectively) and the top twenty semantic features recording the largest principal component loading values. Most notably, the second fMRI noun for MiF-PC3 with the most sex-related connotation is also “hand”, so the graph shares various semantic contexts pertaining to this effector (body, sex, motions, and hand-carried goods). Bottom: anatomical location of the feature voxels selected from each participant of Mitchell et al. [26] as neural contexts corresponding to those two MiF-PCs. For example, “P1-PC3” denotes feature voxels from the P1 dataset that have neural activation patterns significantly homologous to the principal component vector of MiF-PC3 with respect to the 60 nouns used in the fMRI experiment. These sagittal brain images were smoothed using SPM8 with the full-width at half maximum parameter [3 3 3] to enhance visual effects. The Supporting Information and its figures clarify how to couple a neural component and an MiF-PC using an original fcMRI method applied to this *semantico-neural* paradigm.

Fig 7 shows an example mapping for two circuits (or contexts) in parallel, i.e. conceptual relatedness with extending scope or growing complexity, and unexpectedly widespread fMRI responses to a lexical task, both associated with the nouns ‘bed’ and ‘hand’ (representative words for MiF-PC3: ‘bed’-*HARD-SLEEVE-FINGER-SEX-LINING…* and MiF-PC18: ‘hand’-*CAP-BAG-SHOPPING BAG-WAVE-EXCHANGE…*). Instead of determining some categorically-classified semantic atlas on the cortex (like “*furniture*” for ‘bed’ and “*body parts*” for ‘hand’), we generate a binding of informative voxels as a “*neural context*” (similar to a “*semantic space*” [71]), which serves as a counterpart to a lexical mapping of a key noun together with its semantic features. Note that all of these words are treated via fine-grained serial information as freely associated concepts under MiF-based principal components (extended to connotations such as sex, motions, and hand-carried goods; see the third column of S1 Table) that are intricate, context-sensitive, and in some way systematic.

Although the free association norms gathered in a thesaurus reflect the social, cultural, and linguistic backgrounds of the informants who contributed to the data collections [12], the consequent attenuation of individual traits is a common and ineluctable process in data compilation. However, our modelling of double-articulated components enables us to extract individual variability (or, as it were, idiosyncrasies in fMRI responses) from such a synthesised and averaged dictionary, through the biased correlation between MiF-PCs and relevant informative voxels. For example, in the case of P1, we can recognise a sort of *signature pattern* in that 46.2% of feature-voxels (161 out of 348, see the first column of S2 Table) form a wide range of neural context exclusively mediated by MiF-PC3, which is biased towards various sexual implications.

Individuals differ markedly in terms of the location of voxels sensitive to each MiF-PC. It is worth noting that some neural contexts, such as the voxels taken from P4 corresponding to MiF-PC3 (and P8 sensitive to MiF-PC44, see S1 Text, Section II with S4 Fig), can be interpreted by *simulation semantics* [72–76], which is a field of linguistics embodiment theory [77–88]. An intriguing consistency emerges, if only partially, between *areas* and *meanings*, with a relationship prescribed as *somatomotor* or *somatosensory* in the literature of functional anatomy. In fact, P4’s voxels related to MiF-PC3 show that, as if connoting some rehearsal of previous tangible (perceptual-motor) experiences, the Left Precentral, Left Superior Frontal Lobe, Left Supplementary Motor Area, Left Inferior Parietal Lobe, etc., are coupled with some of the most constitutive semantic features with the largest principal component loadings, such as “*HARD*”, “*SLEEVE”*, “*FINGER*”, “*SEX*”, “*FEET*”, “*BODY*”, “*REACH*”, and “*WRIST*”.

However, as such a finding is somewhat narrow, our modelling must be considered as no more than preliminary; delineating an exact parallel map between a neural circuit and semantic network remains a task for future research, at least for a robust signature of an individual subject. We are not yet in a position to introduce any full-fledged *hodological* view into a semantico-anatomical distributional representation in the context of computational neurolinguistics. Similarly, we cannot argue that, for instance, expanding conceptual associations could gain contiguous neural resources as a clearly articulated counterpart. The overlaid components based on our fcMRI-like modelling merely create a chain of fully connected complete graphs on the neural side. Whether already-known anatomical networks underlie the neural contexts that bundle selected voxels that are sensitive to particular concepts remains an open question. However, the results shown in the Supporting Information demonstrate that the most-watched fcMRI anatomical areas frequently emerge in neural contexts, such as the Extrastriate Cortex with Fusiform, Middle Occipital Gyrus, Lingual, and Precuneus (see S1 Text, S2, S3 and S5 Figs) [89].

### Conclusions

In this article, we have proposed a novel distance definition for a graph. This Markov-inverse-F measure (MiF) exploits both geodesic information and the co-occurrence adjustment. By applying our new similarity coefficient to complex networks built from word association norms (EAT), we created a set of intermediate semantic features and their coupling weights for predicting the neural responses to words. In spite of a size constraint, our MiF-based decoding model allowed us to predict, in the wake of Mitchell et al. [26], but using conceptual associations with various interpretations, the neural response to each unknown word with better predictive accuracy than other decoding models based on conventional similarity coefficients.

Moreover, those voxels most responsive to a particular concept were extracted as members of a neural context by leveraging a basic idea of fcMRI. We briefly described the formation of this neural context in terms of the MiF-based principal components as the most overarching and informative semantic features. Although, at the single subject level, we found some cases that seem to embody the physiological process of cognition, large individual differences were observed in the location and scope of neural contexts as a modality of distributional representation. Further challenges will aim to elucidate the mutual relationship between semantic and neural networks as two layers in a globally unified space of computational neurolinguistics.

## Supporting Information

S1 Text Details on MiF-based neuro-computational networks. (DOCX)

S1 Fig Nine bipartite graphs between the 59 MiF principal components and the 500 selected voxels. These graphs were obtained for participants P1–P9 of Mitchell et al. [26] between i) the corpus-related set of 59 MiF principal components (numbered circles on the left) from the EAT dataset, and ii) the participant-wise brain-related set of 500 top voxels (feature voxels) selected by ANOVA (circles on the right), both in terms of the 60 nouns used as stimulus items in the fMRI experiments. Nodes on either side with *r* values greater than 0.330104 are connected. This figure was created using Mathematica 8. We can see that some MiF-PC hubs are linked to many selected voxels, but the pattern is different for each participant. For example, 46.2% of the feature voxels collected from P1 form a wide range of neural context exclusively mediated by MiF-PC3.
(TIF)

S2 Fig Semantic adjacency graphs (top) corresponding to the largest neural contexts (bottom). These contexts were built from the P1–P9 datasets of Mitchell et al. [26]. Isolated nodes have been removed. The series of sagittal slices for mapping the feature voxels of the largest neural context in the standard brain was smoothed using SPM8 with the full-width at half maximum parameter of [3 3 3] to enhance visual effects. We can see that the core neural contexts (largest components) tend to produce bead-like shapes, and encompass a wide range of areas with conspicuous variability across participants.
(TIF)

S3 Fig Semantic and neuro-anatomical adjacency graphs (top) and mapping of their neural contexts (bottom) from participant P2. Components C1–C5 illustrate the conceptual relatedness within each MiF-PC and the selected feature-voxel networks that it sustains and overlays in the space of computational neurolinguistics. For the AAL notation, refer to S3 Table. These neural contexts are either global bead-like networks, large but local networks, or purely local fully connected graphs. The distribution of important voxels in P2 tends to be biased toward the Extrastriate Cortex and its peripheral areas.
(TIF)

S4 Fig MiF-PC44 corresponding to a network in the Frontal Lobe of participant P8. MiF-PC44 (‘bicycle’-*TWO-PEDALLER-CLIP-BRAKE-BIKE…*) is mainly composed of nodes located in the Frontal Lobe (such as ‘Frontal_Inf_Tri_L’, ‘Frontal_Mid_L’, ‘Frontal_Sup_L’, ‘Frontal_Sup_Medial_L’, and so on). Some of these voxels are extracted from regions (Brodmann areas 6, 8, and 9) connected to executive functions with visual control, which is a favourable phenomenon for simulation semantics in embodiment theory. For the AAL notation, refer to S3 Table.
(TIF)

S5 Fig Neural contexts with multiple feature voxels mediated by MiF-PCs 3 and 18. MiF-PC3 represents the series (‘bed’-*HARD-SLEEVE-FINGER-SEX-LINING…*) and MiF-PC18 denotes (‘hand’-*CAP-BAG-SHOPPING BAG-WAVE-EXCHANGE…*). These were examined in the section on MiF-based neuro-computational networks in the main text. “P*i*-PC*j*” denotes feature voxels in dataset P*i* that exhibit neural activation patterns significantly homologous to the principal component vector of MiF-PC*j*. This figure shows adjacent clusters sharing at least one feature voxel. Other MiF-PCs adjacent to PC 3 or 18 in a participant-wise neural context are abbreviated in this figure, with the most contributive fMRI noun with the largest principal component score illustrated, such as PC6-fly. We can glimpse an interlocking scheme between conceptual association and neural response in P4-PC3, where the perceptuo-motor simulation postulated by embodiment theory (see the main text) is linked with MiF-PC59 (‘closet’-*CUPBOARD-WARDROBE-SPACE-LOVE-WHITE…*).
(TIF)

S1 Table Information on MiF-PCs given by top two PC scores and top twenty PC loadings. This file should be used with the name “Information on MiF-PCs through the top two PC scores and the top twenty PC loadings.csv”.
(CSV)

S2 Table Participant-wise number of feature-voxels mediated by each MiF-PC. This file should be used with the name “Participant-wise number of feature-voxels mediated by each MiF-PC.csv”.
(CSV)

S3 Table Abbreviations for AAL list. This file should be used with the name “Abbreviations for AAL.csv”.
(CSV)

S1 Dataset Adjacency matrix (under the format of *matrix market*) of EAT. This file should be used with the name “adjacencyMatrix.mtx”. Subgraph extracted from EAT for connecting the 60 fMRI stimulus nouns used by Mitchell et al. [26].
(MTX)

S2 Dataset Vertex labels of S1 Dataset. This file should be used with the name “vertexLabels.csv”.
(CSV)

S3 Dataset MiF distance matrix computed from the semantic network of EAT. This file should be used with the name “MiFdistanceMatrix.csv”. Rows: 60 fMRI stimulus nouns; Columns: the 836 in-between words with degree greater than 5.
(CSV)

S1 Program Mathematica script for the computation of MiF, Jaccard, and Simpson coefficients between any pair of vertices in a network given as a sparse array. This file should be used with the name “MiF.m”.
(M) Click here for additional data file.

S2 Program Mathematica script for evaluating graph clustering results obtained from MiF with different *β* values, Jaccard, Simpson, and cosine similarity. (PDF)
