# Supplementary material for: Using Graph Components Derived from an Associative Concept Dictionary to Predict fMRI Neural Activation Patterns that Represent the Meaning of Nouns
Source: PLoS One. 2015 Apr 30;10(4):e0125725. doi: 10.1371/journal.pone.0125725 (PMC4482269; doi:10.1371/journal.pone.0125725)
Supplement: S1 Text — (DOCX) [file pone.0125725.s001.docx]

**Supporting Information Text S1. Details on MiF-based neuro-computational networks.**

**Section I. Bipartite fcMRI**

In our graph-theoretical approach, the integration of linguistic and physiological networks, as mentioned in the main paper (Results and Discussion: MiF-based neuro-computational networks), would take the form of a bipartite graph that could be subdivided into two disjoint sets representing documentary and neuro-imaging information. Within each set, there is no inner link between independent members, but a bridging edge connects one vertex in the corpus-related set with the other in the brain-related set with some adjusted probability. A variant of fcMRI analysis was thus performed by embedding—through a bipartite graph architecture—the semantic associative network (on the corpus side) into the neuro-linguistic signal configuration (on the fMRI side). We take advantage of pattern homology between the two different fields to place the voxels together in a context, instead of computing, as is usually the case with fcMRI, the time series correlation of any pair of voxels to describe the adjacency relationship.

In more detail, this “*bipartite* fcMRI” method was applied to the following two disjoint sets: 1) the principal components extracted as semantic features from the connectivity strengths measured by MiF similarity for the co-occurrence data of the EAT word association norms (i.e. “MiF-PCs”); 2) the mean intensity values of the machine-learning features selected as the most informative voxels (i.e. top f-values from ANOVA) for the property generation task with respect to fMRI stimulus nouns (i.e. “feature-voxels”).

We computed the overall pairwise Pearson correlations between one vector from the 59 MiF-PCs (removing the 60th principal component for its incommensurable smallness) and another from the 500 feature-voxels (following [26] and the conventional criteria for the number of features selected in MVPA [64]). Because the dimension of each vector corresponds to the number of fMRI nouns (60), we set the threshold *r* value to 0.330104 (p < 0.01, according to the no-correlation test) to create a 59 × 500 binary matrix $X(x_{\mathrm{ij}})$ in which $x_{\mathrm{ij}}$ is set to 1 if $x_{\mathrm{ij}}$ > 0.330104, and to 0 otherwise. The adjacency matrix of the bipartite-graph integrating the two extraneous sets was produced as $A=\left(\begin{matrix} O & X \\ X^{t} & O \end{matrix}\right)$. Supporting Information Figure S1 shows the bipartite graphs computed for the nine participants (P1-P9). In every case, some vertices on the subgraph of the MiF-PCs recorded outlier degrees and could be discerned as hubs, but these were different for each participant.

To further analyse the MiF-PC hubs and mediate the feature-voxels in the neural space, we created the participant-wise graphs $V(v_{ij})=A^{t}A$, where $v_{ii}=0$, and *v_ij_* is set to 1 if . This sheds light on the functional relatedness of the feature voxels, and especially their labelled anatomical regions. This brain network was based on the adjacency of feature voxels via the MiF-PCs, playing the role of edges this time. Because of the well-matched distribution of MiF-PC hubs and MiF-PC vertices with moderate degrees on the semantic side of this bipartite graph, both distributed (global) and clustered (local) representations could be obtained as separate fcMRI connected components of feature voxels. We describe such clusters or successions of cluster units as a “neural context”.

**Section II. Participant-wise neural context**

We could extract one or two major neural contexts from each participant, broadly encompassing various anatomical regions across the brain. Each large neural context in the voxel space is underpinned by MiF-PC hubs, now converted into its many edges. By virtue of the MiF-PC hubs’ plurality at the participant level (except in the case of participant P3), we have a *bead-like* link between multiple components, each of which is inherently a complete graph. For the sake of illustration, we focus on the largest neural contexts built from the individual datasets of P1-P9.

The upper panel of Supporting Information Figure S2 was created using Mathematica 8 to express the MiF-PC constituents of the largest neural connected components in graph form. The vertices in each subgraph are the representative fMRI nouns and the semantic features having the largest principal component score and largest loading values, respectively. These graphs are parts of the semantic network (Supporting Information Figure 2 in the main paper) of the EAT dataset. The lower panel of Supporting Information Figure S2 shows the abstract shapes of the largest neural contexts as global feature-voxel networks in an intuitively recognisable way. Together with the XjView toolbox for SPM (http://www.alivelearn.net/xjview8/), we can situate the selected feature-voxels that are members of the largest neural context inside the normalised brain for each participant.

To enable a participant-wise analysis on the relationship between the semantic networks and neural contexts, Supporting Information Figure S3 indicates the adjacency graphs (or connected components) representing the P2 dataset. The graphs were extracted to show the semantic relatedness within each MiF principal component and the feature-voxel connectivity that it sustains and overlays within what we term the computational neurolinguistics space. The upper panel of Supporting Information Figure S3 was produced from the aforementioned bi-partite graphs between the linguistic and neural datasets (see Supporting Information Figure S1), and the lower one was created to show the location of the 500 feature voxels within the standardised brain template. All isolated vertices in the semantic subgraphs were removed from Supporting Information Figure S3, and the same applies to all small neural components with fewer than five vertices. The anatomical labels are based on the Automated Anatomical Labeling (AAL) atlas [88].

As shown in Supporting Information Figure S3, five neural contexts from the P2 dataset were treated. These were the global networkC1, a large but in some way local network C2, and the local components C3–C5 that form complete graphs. Interestingly, the core neural contexts are characterised by the long linking of distant anatomical regions. Seen as distant locations with occasionally recognition between vertices with an identical label (for example, as denoted in Supporting Information Figure S3, there are four steps in C2 between one ‘Lingual_right’ (LR) vertex and another LR node), it would be natural to interpret this global involvement of areas not as evidence for physically existent brain networks, but as proof for an extended neural context, that is, the broadened scope of a synchronous signal modulation.

To be more specific, C1 is sustained by MiF-PCs 18, 32, 44, and 46, and connects the Left Cerebellum, Temporal, Occipital, and Parietal Lobes of both hemispheres, and even motor areas in the Frontal Lobe. The first fMRI noun and the top 10 semantic features in EAT recording the largest score and largest loading values for each of these principal neural contexts are as follows: MiF-PC18: ‘hand’ *-CAP -BAG -SHOPPING BAG -WAVE -EXCHANGE...*; MiF-PC32: ‘saw’ -*EYES -HEARD -WATCHED -HER -CIRCULAR...*; MiF-PC44: ‘bicycle’ -*TWO -PEDALLER -CLIP -BRAKE -BIKE...*; MiF-PC46: ‘refrigerator’ -*FREEZE -COLDER -SNOW -ICE -OTTER..*. Subsequently, network C2 consists of beads MiF-PC1 (‘train’ -*RAIL -TRAVEL -OMNIBUS -ENGINE -BUS...*), MiF-PC49 (‘arch’ -*BRIDGE -BUILDING -ENEMY -CIRCLE -MARBLES...*), and MiF-PC59 (‘closet’ -*CUPBOARD -WARDROBE -SPACE -LOVE -WHITE...*), which cover a wide area of the Occipital division around the Calcarine Sulcus.

In contrast to these chains of MiF-PCs and brain areas, each separate neural context with a small number of vertices was generated by a single MiF-PC that fully connects the feature voxels as a cluster in terms of anatomical vicinity or continuity (except for some exclaves, such as ‘Parietal_Inf_R’ in C5 for participant P2). In these constellations, there is an almost one-to-one correspondence between a single MiF-PC and a sphere of local regions. For example, C3 given by MiF-PC36 (‘arm’ -*DRILL -SORE -RINGWORM -BREAKFAST -DIARY...*), C4 given by MiF-PC40 (‘spoon’ -*FULL -CORNFLAKES -MOON -LEGS -PUDDING...*), and C5 given by MiF-PC4 (‘window’ -*OPEN -PICTURE -BUILDINGS -BOX -SMASH...*) for participant P2 tend to pack determinate regions in the posterior part of the brain. However, there is also some individual variability in the location of such clusters: one small neural context derived from subject P8 by MiF-PC44 (‘bicycle’ -*TWO -PEDALLER -CLIP -BRAKE -BIKE...*) was mainly composed of nodes in the Frontal Lobe, unlike for P2 (cf. Supporting Information Figure S4), where the neural response to the same MiF-PC is lateralised to the visual area.

As for the individual variability in neural contexts, the main paper treated the example of MiF-PCs 3 (‘bed’ *-HARD -SLEEVE -FINGER -SEX -LINING*...) and 18 (‘hand’ *-CAP -BAG -SHOPPING -BAG -WAVE -EXCHANGE*...). Supporting Information Figure S5 displays the participant-wise network structures of neural contexts, giving a full account of the overlay of semantic and neural spaces shown in Supporting Information Figure 5 of the main paper. As previously discussed, the conceptual paradigm associated with body, sex, motions, and belongings has a counterpart in the different types of neural context given by various hub distributions in bipartite fcMRI.

As a result, coupling the feature voxels through the set of lexical factors in the bipartite fcMRI allows us to substantiate more general information about the world of conceptual processing than when semantic features are limited to a particular lexicon. A semantic entity computed from a word association network could be fitted into a subgraph or cluster of another complex network that bundles neural units with a similar information pattern to a concept or meaning (not a single word). Such a correspondence between semantic and neural entities is not exclusive to a fixed and localised injective mapping, but, through the overlapping words among the sub-networks that they could represent, MiF-PCs enable us to graft different brain areas as loci for various unfolded meanings.
