# Supplementary figures and images for: Using Graph Components Derived from an Associative Concept Dictionary to Predict fMRI Neural Activation Patterns that Represent the Meaning of Nouns
Source: PLoS One. 2015 Apr 30;10(4):e0125725. doi: 10.1371/journal.pone.0125725 (PMC4482269; doi:10.1371/journal.pone.0125725)

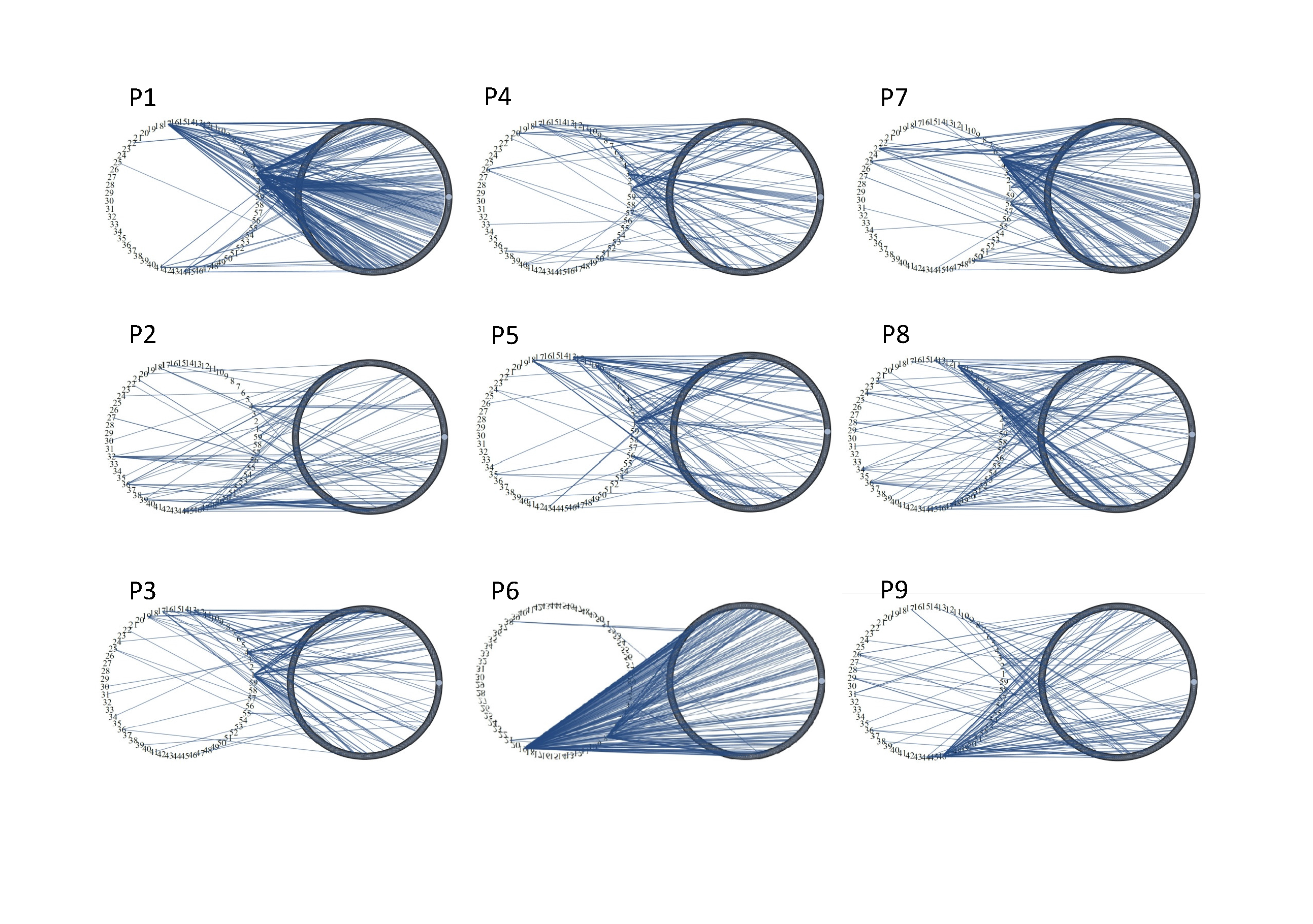

Supplement: S1 Fig — These graphs were obtained for participants P1–P9 of Mitchell et al. [26] between i) the corpus-related set of 59 MiF principal components (numbered circles on the left) from the EAT dataset, and ii) the participant-wise brain-related set of 500 top voxels (feature voxels) selected by ANOVA (circles on the right), both in terms of the 60 nouns used as stimulus items in the fMRI experiments. Nodes on either side with r values greater than 0.330104 are connected. This figure was created using Mathematica 8. We can see that some MiF-PC hubs are linked to many selected voxels, but the pattern is different for each participant. For example, 46.2% of the feature voxels collected from P1 form a wide range of neural context exclusively mediated by MiF-PC3. (TIF) [file pone.0125725.s002.tif]

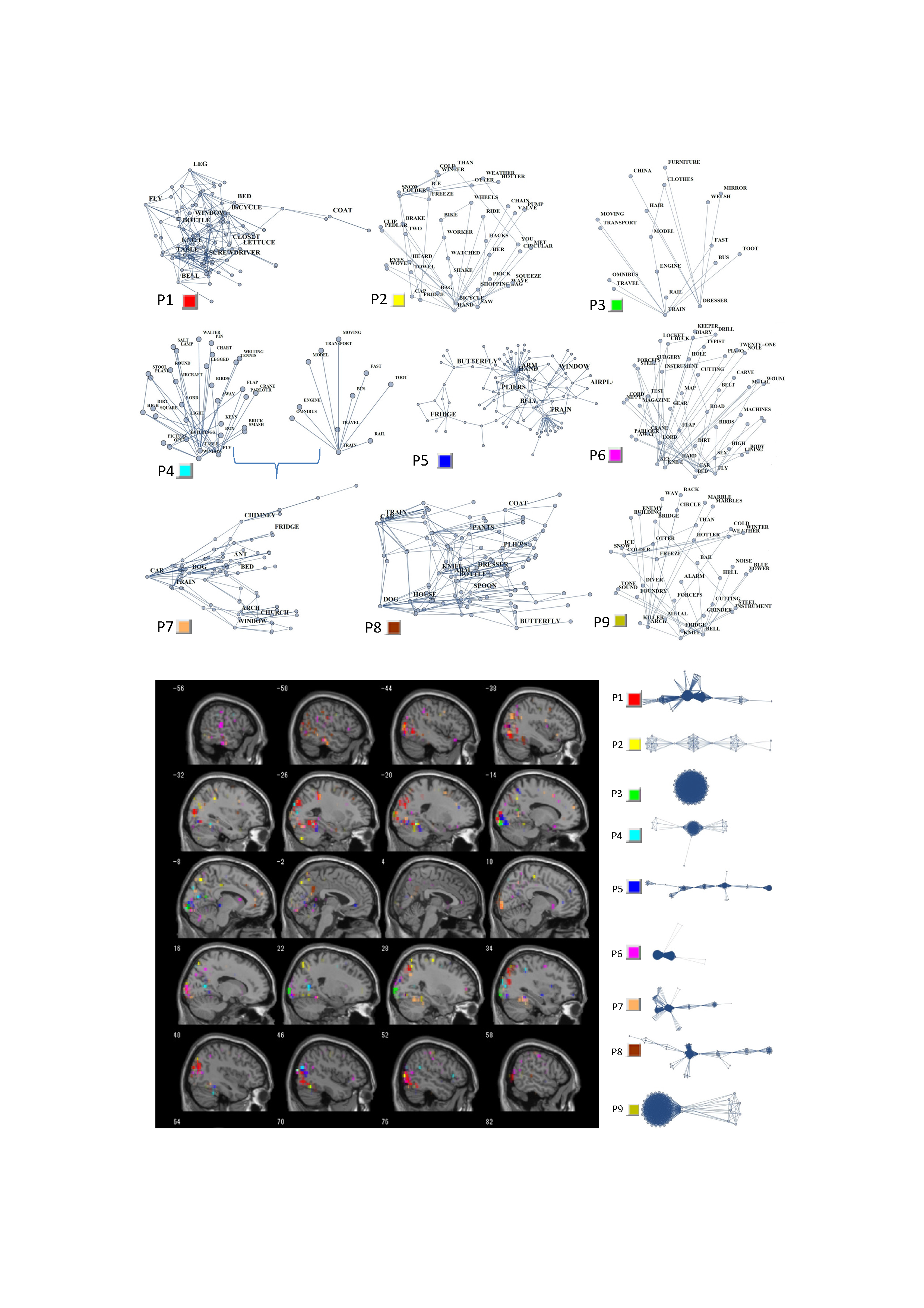

Supplement: S2 Fig — These contexts were built from the P1–P9 datasets of Mitchell et al. [26]. Isolated nodes have been removed. The series of sagittal slices for mapping the feature voxels of the largest neural context in the standard brain was smoothed using SPM8 with the full-width at half maximum parameter of [3 3 3] to enhance visual effects. We can see that the core neural contexts (largest components) tend to produce bead-like shapes, and encompass a wide range of areas with conspicuous variability across participants. (TIF) [file pone.0125725.s003.tif]

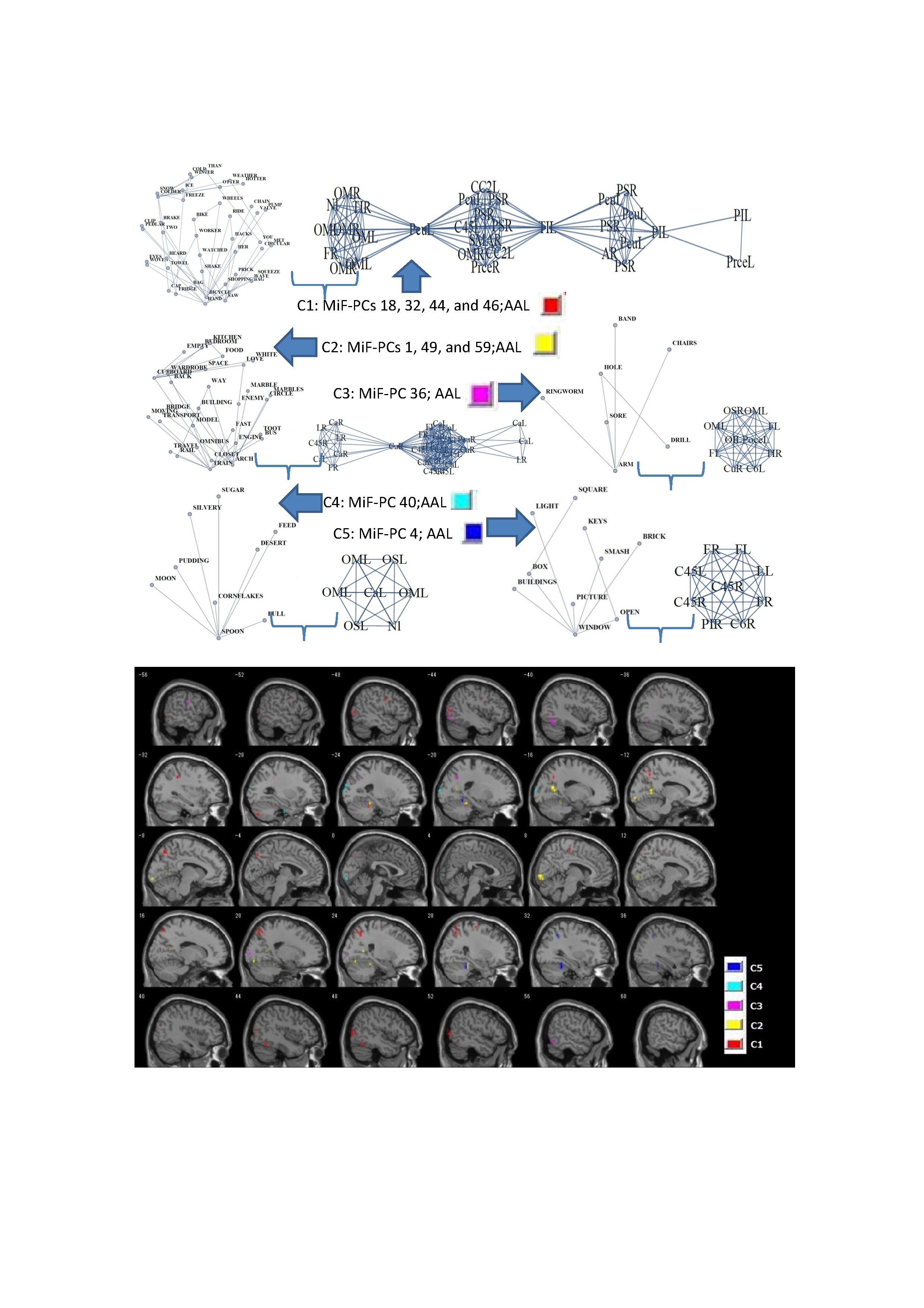

Supplement: S3 Fig — Components C1–C5 illustrate the conceptual relatedness within each MiF-PC and the selected feature-voxel networks that it sustains and overlays in the space of computational neurolinguistics. For the AAL notation, refer to S3 Table. These neural contexts are either global bead-like networks, large but local networks, or purely local fully connected graphs. The distribution of important voxels in P2 tends to be biased toward the Extrastriate Cortex and its peripheral areas. (TIF) [file pone.0125725.s004.tif]

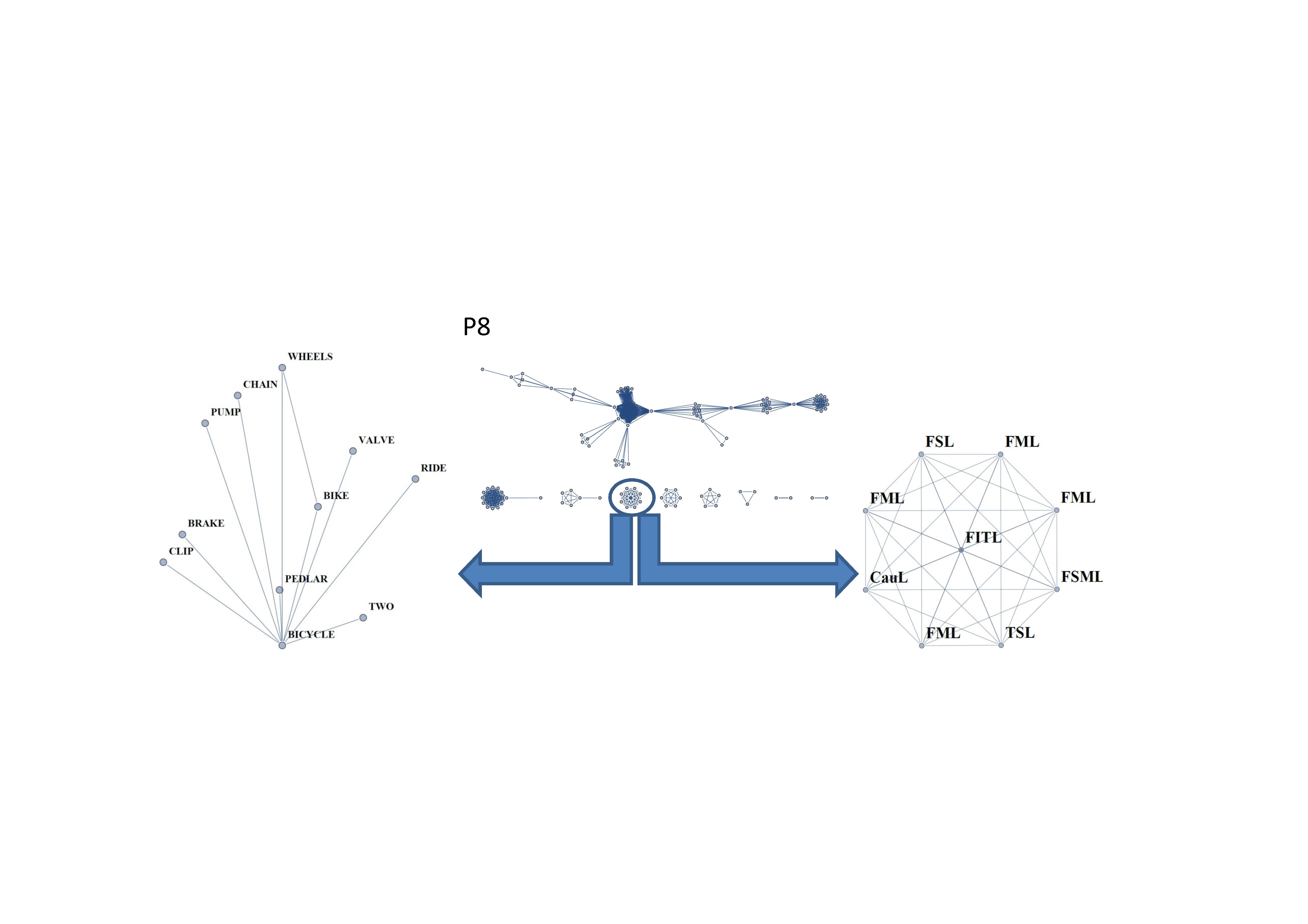

Supplement: S4 Fig — MiF-PC44 (‘bicycle’-TWO-PEDALLER-CLIP-BRAKE-BIKE…) is mainly composed of nodes located in the Frontal Lobe (such as ‘Frontal_Inf_Tri_L’, ‘Frontal_Mid_L’, ‘Frontal_Sup_L’, ‘Frontal_Sup_Medial_L’, and so on). Some of these voxels are extracted from regions (Brodmann areas 6, 8, and 9) connected to executive functions with visual control, which is a favourable phenomenon for simulation semantics in embodiment theory. For the AAL notation, refer to S3 Table. (TIF) [file pone.0125725.s005.tif]

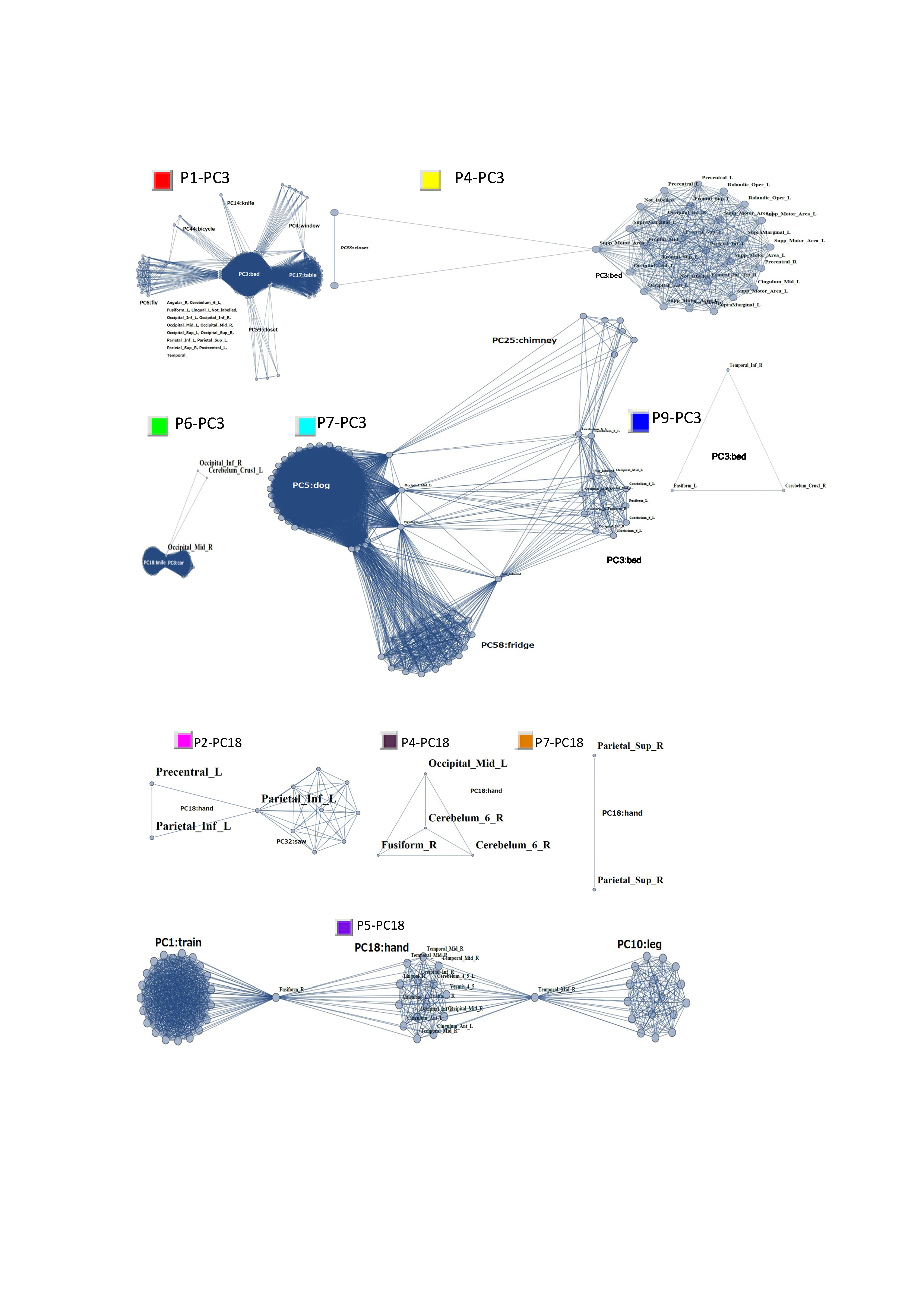

Supplement: S5 Fig — MiF-PC3 represents the series (‘bed’-HARD-SLEEVE-FINGER-SEX-LINING…) and MiF-PC18 denotes (‘hand’-CAP-BAG-SHOPPING BAG-WAVE-EXCHANGE…). These were examined in the section on MiF-based neuro-computational networks in the main text. “Pi-PCj” denotes feature voxels in dataset Pi that exhibit neural activation patterns significantly homologous to the principal component vector of MiF-PCj. This figure shows adjacent clusters sharing at least one feature voxel. Other MiF-PCs adjacent to PC 3 or 18 in a participant-wise neural context are abbreviated in this figure, with the most contributive fMRI noun with the largest principal component score illustrated, such as PC6-fly. We can glimpse an interlocking scheme between conceptual association and neural response in P4-PC3, where the perceptuo-motor simulation postulated by embodiment theory (see the main text) is linked with MiF-PC59 (‘closet’-CUPBOARD-WARDROBE-SPACE-LOVE-WHITE…). (TIF) [file pone.0125725.s006.tif]
