# Supplementary material for: Using Graph Components Derived from an Associative Concept Dictionary to Predict fMRI Neural Activation Patterns that Represent the Meaning of Nouns
Source: PLoS One. 2015 Apr 30;10(4):e0125725. doi: 10.1371/journal.pone.0125725 (PMC4482269; doi:10.1371/journal.pone.0125725)
Supplement: S2 Program — (PDF) [file pone.0125725.s014.pdf]

```
In[59]:= Needs["HierarchicalClustering`"];
karate = Import["karate-2.mtx"]

Out[60]= SparseArray[<156>, {34, 34}]

In[61]:= TakePartTwo[list_] := Part[#, 2] & /@ list;
DegreesFromSparseAdjacencyMatrix[sparseadjacencymatrix_] :=
  Module[{sparselength = {}, ars = {}, nodeweight = {}},
   sparselength = Length[sparseadjacencymatrix];
   ars = ArrayRules[sparseadjacencymatrix] // Most;
   nodeweight = Split[{#[[1]][[1]], #[[2]]} & /@ ars];
   Apply[Plus, #] & /@ (TakePartTwo[#] & /@ nodeweight)];
GetZeroRowsAndColumnsFromSparseAdjacencyMatrix[samplesparsearray_] :=
  Module[{sparselength = {}, ars = {}, tmp = {}, linkpairs = {}, nonzerorow = {}},
   sparselength = Length[samplesparsearray];
   ars = ArrayRules[samplesparsearray];
   tmp = Cases[ars, x : ({_, _} → 1)];
   linkpairs = ReplaceAll[tmp, ({x_, y_} → 1) → {x, y}];
   nonzerorow = First[#] & /@ Split[Sort[Flatten[linkpairs]]];
   Complement[Range[sparselength], nonzerorow]];
DegreesFromSparseAdjacencyMatrixNew[sparsearray_] :=
  Module[{poslist, mylist, deg},
   poslist = GetZeroRowsAndColumnsFromSparseAdjacencyMatrix[sparsearray];
   deg = DegreesFromSparseAdjacencyMatrix[sparsearray];
   If[GetZeroRowsAndColumnsFromSparseAdjacencyMatrix[sparsearray] == {},
    deg, (For[j = 1; mylist[1] = deg, j ≤ Length[poslist], j++,
      mylist[j + 1] = Insert[mylist[j], 0, poslist[[j]]]]; mylist[j])]];
Jaccard[adjacencymatrix_, x_, y_, gamma_] := Module[{poweradjmatrix, poweradjcheck,
    deglist, numnodes, spstep, jaccard}, k = 1; tmat[1] = adjacencymatrix;
   poweradjmatrix[1] = tmat[1] ; While[k ≤ gamma - 1,
    tmat[k + 1] = tmat[k].tmat[1]; k++];
   poweradjmatrix = Table[1, {i, 1, gamma}];
   For[i = 1, i ≤ gamma, i++, poweradjmatrix[[i]] = tmat[i]];
   poweradjcheck = poweradjmatrix[[#, x, y]] & /@ Range[gamma];
   deglist = DegreesFromSparseAdjacencyMatrixNew[adjacencymatrix];
   numnodes = Max[Most[#[[1]] & /@ ArrayRules[adjacencymatrix]]];
   spstep =
    If[First[poweradjcheck] == 1, 1, First[Flatten[Position[poweradjcheck, First[
          Complement[poweradjcheck, Table[0, {i, 1, Length[poweradjcheck]}]]]]]]];
   jaccard = If[spstep == 1, 1 / (deglist[[x]] + deglist[[y]]) // N,
     poweradjmatrix[[spstep, x, y]] /
       (Plus @@ (poweradjmatrix[[spstep, x, #]] & /@ Range[numnodes]) + Plus @@
          (poweradjmatrix[[spstep, y, #]] & /@ Range[numnodes])) // N]];
MiF[adjacencymatrix_, x_, y_, beta_, gamma_, coefficientlist_] :=
  Module[{alphalist, matpower, matpowerxandy, sumupx, sumupy, numerator, denominator,
    val = 0}, alphalist = Table[N[(1 / coefficientlist[[gamma]]) ^i], {i, 1, gamma}];
   Clear[i]; Clear[k];
   k = 1; tmat[1] = adjacencymatrix;
   While[k ≤ gamma - 1, tmat[k + 1] = tmat[k].tmat[1]; k++];
   For[i = 1, i ≤ gamma, i++, matpower = tmat[i];
    matpowerxandy = matpower[[x, y]]; sumupx = Plus @@ matpower[[x]];
    sumupy = Plus @@ matpower[[y]];
    numerator =
     N[alphalist[[i]] * matpowerxandy * (beta * sumupx + (1 - beta) * sumupy)];
    denominator = sumupx * sumupy;
    val = val + N[(numerator / denominator)]];
```

```
    val];
Simpson[adjacencymatrix_, x_, y_, gamma_] := Module[{poweradjmatrix, poweradjcheck,
     deglist, numnodes, spstep, degx, degy, smallerdeg, simpson}, k = 1;
    tmat[1] = adjacencymatrix; poweradjmatrix[1] = tmat[1] ;
    While[k ≤ gamma - 1, tmat[k + 1] = tmat[k].tmat[1]; k++];
    poweradjmatrix = Table[1, {i, 1, gamma}];
    For[i = 1, i ≤ gamma, i++, poweradjmatrix[[i]] = tmat[i]];
    poweradjcheck = poweradjmatrix[[#, x, y]] & /@ Range[gamma];
    deglist = DegreesFromSparseAdjacencyMatrixNew[adjacencymatrix];
    numnodes = Max[Most[#[[1]] & /@ ArrayRules[adjacencymatrix]]];
    spstep =
     If[First[poweradjcheck] == 1, 1, First[Flatten[Position[poweradjcheck, First[
          Complement[poweradjcheck, Table[0, {i, 1, Length[poweradjcheck]}]]]]]]];
    degx = Plus @@ (poweradjmatrix[[spstep, x, #]] & /@ Range[numnodes]);
    degy = Plus @@ (poweradjmatrix[[spstep, y, #]] & /@ Range[numnodes]);
    smallerdeg = If[degx < degy, degx, degy];
    simpson = If[spstep == 1, 1 / smallerdeg // N,
      poweradjmatrix[[spstep, x, y]] / smallerdeg // N]];
CosineDist[adjacencymatrix_, x_, y_] :=
  N[(adjacencymatrix[[x]].adjacencymatrix[[y]]) /
     (Norm[adjacencymatrix[[x]]] * Norm[adjacencymatrix[[y]]])];
MyClusterFlattenTwoLeaves[directAgglomerateResult_] :=
  {Sort[ClusterFlatten[directAgglomerateResult[[1]]]],
    Sort[ClusterFlatten[directAgglomerateResult[[2]]]]};
trueclus = {{1, 2, 3, 4, 5, 6, 7, 8, 11, 12, 13, 14, 17, 18, 20, 22},
    {9, 10, 15, 16, 19, 21, 23, 24, 25, 26, 27, 28, 29, 30, 31, 32, 33, 34}}
    (*True clusters of Karate Club.*);
```

```
In[22]:= comb = Select[Tuples[{Range[34], Range[34]}], #[[1]] != #[[2]] &];
```

```
In[71]:= MiFdist = MiF[karate, #[[1]], #[[2]], 0.5, 6,
      {1, 1.618033988749895, 1.8392867552141607, 1.9275619754829254,
       1.9659482366454855, 1.9835828434243263, 1.9919641966050354,
       1.9960311797354144, 1.9980294702622872, 1.9990186327101014}] & /@ comb;
(*beta value id 0.5*)
Jaccarddist = Jaccard[karate, #[[1]], #[[2]], 6] & /@ comb;
Simpsondist = Simpson[karate, #[[1]], #[[2]], 6] & /@ comb;
Cosinedist = CosineDist[karate, #[[1]], #[[2]]] & /@ comb;
MiFdist001 = MiF[karate, #[[1]], #[[2]], 0.01, 6,
      {1, 1.618033988749895, 1.8392867552141607, 1.9275619754829254,
       1.9659482366454855, 1.9835828434243263, 1.9919641966050354,
       1.9960311797354144, 1.9980294702622872, 1.9990186327101014}] & /@ comb;
(*beta
 value
 is
 0.01!*)
```

In[76]:=
```
MiFdistmat = MapThread[#1 → #2 &, {comb, MiFdist}];
Mifdistmatdat = SparseArray[MiFdistmat, {34, 34}];
MiFdistmat05 = DirectAgglomerate[
   (1 / #) & /@ (Normal[Mifdistmatdat] + IdentityMatrix[34]), Linkage → "Ward"];
DendrogramPlot[MiFdistmat05, LeafLabels -> Automatic]
MyClusterFlattenTwoLeaves[MiFdistmat05]
% == trueclus
```

Out[79]=

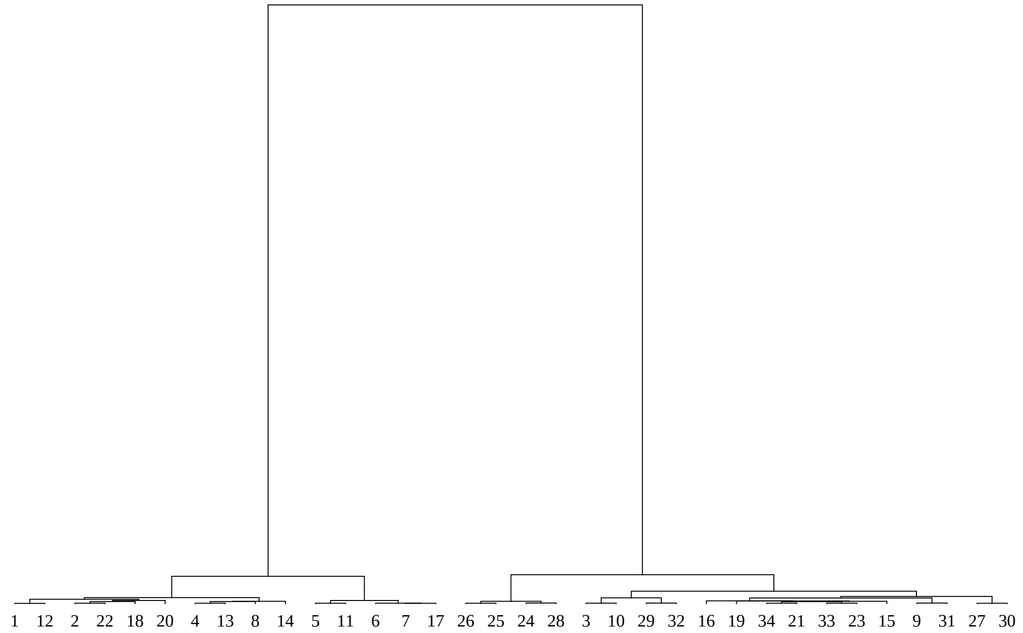

Out[80]=
```
{{1, 2, 4, 5, 6, 7, 8, 11, 12, 13, 14, 17, 18, 20, 22},
 {3, 9, 10, 15, 16, 19, 21, 23, 24, 25, 26, 27, 28, 29, 30, 31, 32, 33, 34}}
```

Out[81]=
```
False
```

In[82]:=
```
Jaccarddistmat = MapThread[#1 → #2 &, {comb, Jaccarddist}];
Jaccarddistmatdat = SparseArray[Jaccarddistmat, {34, 34}];
jacdistmat = DirectAgglomerate[
   (1 / #) & /@ (Normal[Jaccarddistmatdat] + IdentityMatrix[34]), Linkage → "Ward"];
DendrogramPlot[%, LeafLabels -> Automatic]
MyClusterFlattenTwoLeaves[jacdistmat]
% == trueclus
```

Out[85]=

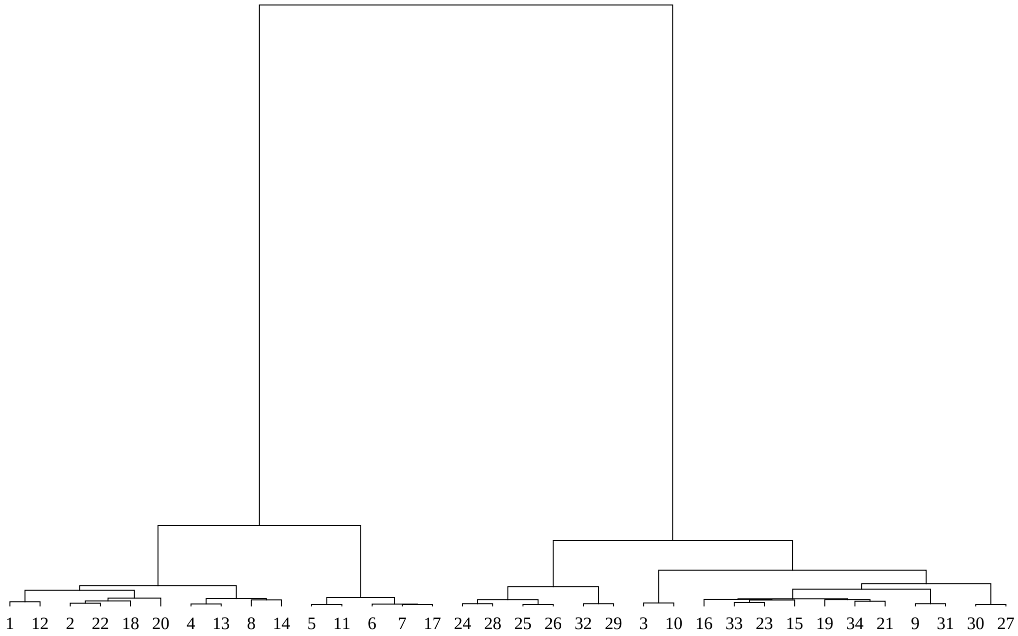

Out[86]= {{1, 2, 4, 5, 6, 7, 8, 11, 12, 13, 14, 17, 18, 20, 22},
{3, 9, 10, 15, 16, 19, 21, 23, 24, 25, 26, 27, 28, 29, 30, 31, 32, 33, 34}}

Out[87]= False

In[88]:=
```
Simpsondistmat = MapThread[#1 → #2 &, {comb, Simpsondist}];
Simpsondistmatdat = SparseArray[Simpsondistmat, {34, 34}];
simpdistmat = DirectAgglomerate[
   (1 / #) & /@ (Normal[Simpsondistmatdat] + IdentityMatrix[34]), Linkage → "Ward"];
DendrogramPlot[%, LeafLabels -> Automatic]
MyClusterFlattenTwoLeaves[simpdistmat]
% == trueclus
```

Out[91]=

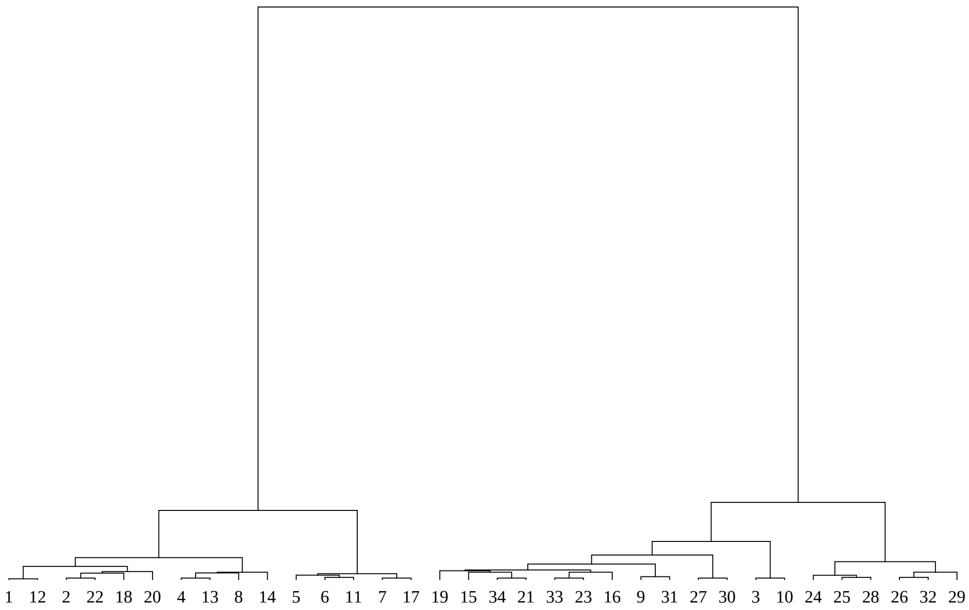

Out[92]=
```
{{1, 2, 4, 5, 6, 7, 8, 11, 12, 13, 14, 17, 18, 20, 22},
 {3, 9, 10, 15, 16, 19, 21, 23, 24, 25, 26, 27, 28, 29, 30, 31, 32, 33, 34}}
```

Out[93]=
```
False
```

In[94]:=
```
Cosinedistmat = MapThread[#1 → #2 &, {comb, Cosinedist}];
Cosinedistmatdat = SparseArray[Cosinedistmat, {34, 34}];
cosdistmat = DirectAgglomerate[(1 / #) & /@
    ((Normal[Cosinedistmatdat + SparseArray[IdentityMatrix[34], {34, 34}]]) /.
      {1. → 1, 0. → 0.01}), Linkage → "Ward"];
(*cosdistmat=DirectAgglomerate[(1/#)&/@
    ((Normal[Cosinedistmatdat+SparseArray[IdentityMatrix[34],{34,34}]])/.
      {1.→1,0.→0}),Linkage→"Ward"];*)
DendrogramPlot[cosdistmat, LeafLabels -> Automatic]
MyClusterFlattenTwoLeaves[cosdistmat]
% == trueclus
```

Out[97]=

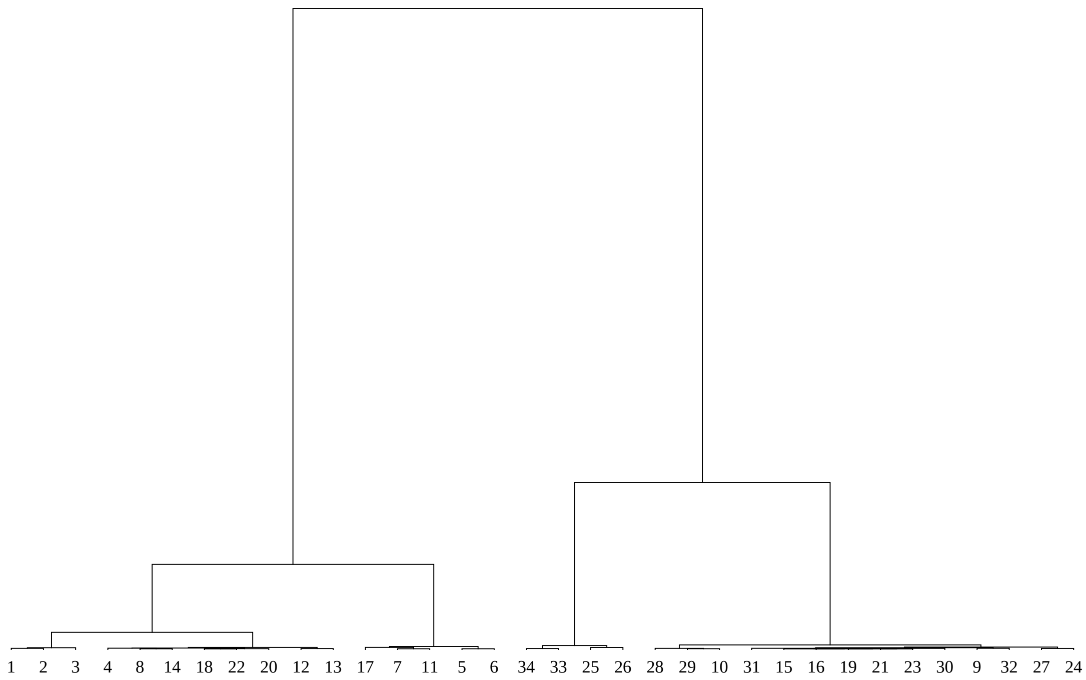

Out[98]=
```
{{1, 2, 3, 4, 5, 6, 7, 8, 11, 12, 13, 14, 17, 18, 20, 22},
 {9, 10, 15, 16, 19, 21, 23, 24, 25, 26, 27, 28, 29, 30, 31, 32, 33, 34}}
```

Out[99]=
```
True
```

In[100]:=
```
MiFdistmat001 = MapThread[#1 → #2 &, {comb, MiFdist001}];
Mifdistmatdat001 = SparseArray[MiFdistmat001, {34, 34}];
damif001 = DirectAgglomerate[
   (1 / #) & /@ (Normal[Mifdistmatdat001] + IdentityMatrix[34]), Linkage → "Ward"];
DendrogramPlot[damif001, LeafLabels -> Automatic]
MyClusterFlattenTwoLeaves[damif001]
% == trueclus
```

Out[103]=

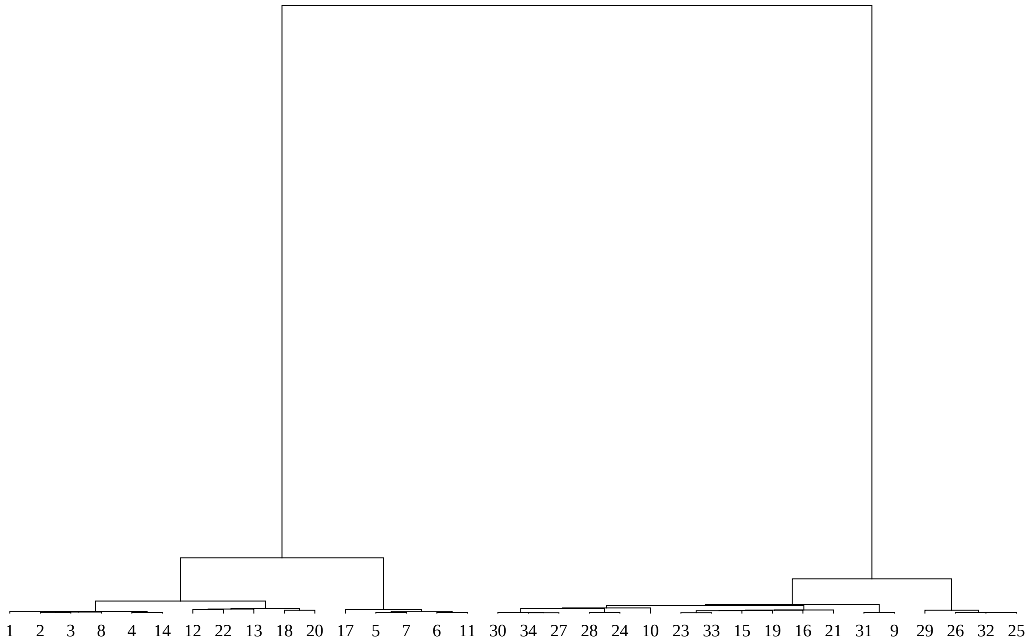

Out[104]=
```
{{1, 2, 3, 4, 5, 6, 7, 8, 11, 12, 13, 14, 17, 18, 20, 22},
 {9, 10, 15, 16, 19, 21, 23, 24, 25, 26, 27, 28, 29, 30, 31, 32, 33, 34}}
```

Out[105]=
```
True
```
